# Metabolomics, Lipidomics, and Antipsychotics: A Systematic Review

**DOI:** 10.3390/biomedicines11123295

**Published:** 2023-12-13

**Authors:** Kyle J. Burghardt, Megan Kajy, Kristen M. Ward, Paul R. Burghardt

**Affiliations:** 1 Department of Pharmacy Practice, Eugene Applebaum College of Pharmacy and Health Sciences, Wayne State University Detroit, Detroit, MI 48201, USA; gh2397@wayne.edu; 2 Department of Clinical Pharmacy, College of Pharmacy, University of Michigan Ann Arbor, Detroit, MI 48109, USA; kmwiese@med.umich.edu; 3 Department of Nutrition and Food Science, Wayne State University Detroit, Detroit, MI 48201, USA; paul.burghardt@wayne.edu

**Keywords:** antipsychotic, metabolomic, lipidomic, human, review

## Abstract

Antipsychotics are an important pharmacotherapy option for the treatment of many mental illnesses. Unfortunately, selecting antipsychotics is often a trial-and-error process due to a lack of understanding as to which medications an individual patient will find most effective and best tolerated. Metabolomics, or the study of small molecules in a biosample, is an increasingly used omics platform that has the potential to identify biomarkers for medication efficacy and toxicity. This systematic review was conducted to identify metabolites and metabolomic pathways associated with antipsychotic use in humans. Ultimately, 42 studies were identified for inclusion in this review, with all but three studies being performed in blood sources such as plasma or serum. A total of 14 metabolite classes and 12 lipid classes were assessed across studies. Although the studies were highly heterogeneous in approach and mixed in their findings, increases in phosphatidylcholines, decreases in carboxylic acids, and decreases in acylcarnitines were most consistently noted as perturbed in patients exposed to antipsychotics. Furthermore, for the targeted metabolomic and lipidomic studies, seven metabolites and three lipid species had findings that were replicated. The most consistent finding for targeted studies was an identification of a decrease in aspartate with antipsychotic treatment. Studies varied in depth of detail provided for their study participants and in study design. For example, in some cases, there was a lack of detail on specific antipsychotics used or concomitant medications, and the depth of detail on sample handling and analysis varied widely. The conclusions here demonstrate that there is a large foundation of metabolomic work with antipsychotics that requires more complete reporting so that an objective synthesis such as a meta-analysis can take place. This will then allow for validation and clinical application of the most robust findings to move the field forward. Future studies should be carefully controlled to take advantage of the sensitivity of metabolomics while limiting potential confounders that may result from participant heterogeneity and varied analysis approaches.

## 1. Introduction

Antipsychotics are an essential component of medication management for the treatment of psychosis and bipolar disorder. However, their use has been steadily increasing in younger patients and for an extended number of disease states, such as depression, autism, conduct disorders, and anxiety [1,2,3]. Antipsychotics work primarily through the dopamine system, and atypical or second-generation antipsychotics also have effects on the serotonin system. Despite being effective medications for many patients, high rates of side effects and non-adherence often lead to multiple trials and even polypharmacy [4,5]. The exact mechanisms by which antipsychotics exhibit their efficacy and side effects remain to be understood, and furthermore, biomarkers that predict antipsychotic outcomes are few.

Investigations into the mechanisms and biomarkers of antipsychotic treatment have been ongoing, and more recently, technological advancements in metabolomics and lipidomics have enabled the simultaneous assessment of tens to hundreds of potential biomarkers in the context of antipsychotic treatment. Metabolomics, the assessment of small molecules in a sample, and lipidomics, the assessment of lipid species in a sample, have rapidly advanced, and with it, the number of investigations reporting their associations with antipsychotic treatment. Despite this growing body of metabolomic and lipidomic literature on antipsychotic treatment, no clear findings have emerged. This may be due to the widely varying metabolomic and lipidomic approaches taken between the studies, which make it difficult to compare findings.

In this systematic review, we aimed to identify and summarize the current literature regarding metabolomic and lipidomic studies of antipsychotic use. The goal of this work is to provide a comprehensive yet succinct summary of the current findings in the area and provide researchers within this field with easily referenced evidence that will allow them to pursue further research that builds off the current evidence base. We also aimed to provide an easily referenced summarization of the field that can be referenced by those interested in specific molecular pathways or metabolites. By providing the current state of the field, the findings here will enable further advancements in antipsychotic metabolomic and lipidomic research by illustrating which metabolites, lipids, and/or pathways show the most promise for future validation and eventual clinical application. The ultimate goal of biomarker use is to improve antipsychotic efficacy, reduce side effects, and improve treatment adherence.

## 2. Materials and Methods

Our systematic review, conducted according to the Preferred Reporting Items for Systematic Reviews and Meta-Analyses (PRISMA) statement guidelines, aimed to identify any reported associations within a metabolomic or lipidomic study with antipsychotic treatment in humans [6]. To that end, Pubmed, Embase, and Web of Science were queried from inception to the time of search (June 2023) using a combination of the following words: antipsychotic, neuroleptic, metabolite, metabolomic, lipid, lipidomic, schizophrenia, bipolar disorder, and individual antipsychotic names. Searches were limited to exclude reviews and exclude non-human investigations. Searches were performed in the “advanced” search toolbox for the databases using Boolean operators and allowing the software to create truncated keywords or Medical Subject Headings (MESH) terms, if available (e.g., antipsychotic keyword input will use variations such as antipsychotics, antipsychotic agent, etc.). Titles and abstracts were first screened, and then those included were screened at the full-text level. Studies were included if they (1) assessed the effect of antipsychotic treatment on metabolomic (or lipidomic) levels via longitudinal methods (i.e., a pre-post study), (2) assessed the effect on antipsychotic treatment on metabolomic levels via correlational methods (e.g., case-control study comparing those on antipsychotics to healthy controls, cross-sectional study looking at correlation between metabolite levels and antipsychotic dose, etc.) and (3) used mass spectrometry or other quantitative technology to assay > 5 metabolites or lipids simultaneously. Studies whose primary aim was to investigate disease-based metabolomic or lipidomic associations (e.g., schizophrenia versus healthy controls) were allowed as long as either all patients were on an antipsychotic (which was compared to a healthy control group) or there was a specific sub-analysis describing an association with antipsychotic treatment such as dosage, antipsychotic type, antipsychotic as a covariate or a comparison between patients that were naive or free of antipsychotics versus patients currently taking antipsychotics. Studies were excluded if they (1) were a review, (2) used non-human populations, or (3) compared antipsychotics to a non-antipsychotic (e.g., antidepressant, mood stabilizer, etc.). Of note, during our exclusion process, we did not identify an NMR-based study that met our inclusion criteria [7]. All screening utilized two authors, and disagreements were resolved through consensus. This systematic review protocol and methods were registered in the Open Science Framework database (https://osf.io/kd2bw?view_only=ee5a1807b390411aa977c58f158f9893, accessed on 6 November 2023).

Data from the included studies were extracted for qualitative synthesis and presentation. The extracted data included study name, authors, population studied, antipsychotics studied, antipsychotic dosage (if available), population age, sex proportion, metabolomic approach (i.e., untargeted versus targeted), number of metabolites or lipids analyzed, and results. Study quality was evaluated using the National Heart, Lung, and Blood Institute (NHLBI) Quality Assessment Tool that is tailored to a given study design. Given the number of included studies and the size of metabolomic studies in general, extracted data was qualitatively summarized in two forms. First, in an abbreviated, reader-friendly form, which is presented in tabular format in the results, and in a more detailed form, which is presented in the supplementary information.

## 3. Results

### 3.1. Description of Included Studies

From the 2876 studies identified as potentially eligible for review, a total of 42 studies were included that investigated the effects of antipsychotics on metabolomic profiles (Table 1) [8,9,10,11,12,13,14,15,16,17,18,19,20,21,22,23,24,25,26,27,28,29,30,31,32,33,34,35,36,37,38,39,40,41,42,43,44,45,46,47,48,49]. The PRISMA flow diagram is included with the supplementary files. Most studies excluded at the full-text stage were removed because they either excluded patients on antipsychotics, the patient group was not all on an antipsychotic, or they did not include a reported association between the metabolomic/lipidomic features and antipsychotics, such as in the studies by Kageyama and Ward [50,51]. Within the Kageyama study, patients were excluded if they were on antipsychotic treatment, while in the Ward study, all patients were on an antipsychotic; however, the metabolomic analysis was performed based on serum insulin quartiles, not antipsychotic dose or type. Twenty-five studies used a pre-post design to analyze the change in metabolites after treatment with an antipsychotic. All pre-post designs were non-randomized, with the exception of one study, which was a randomized controlled trial [30]. The remaining studies were of cross-sectional (10 studies) and case-control (7 studies) design. The primary study population consisted of people with schizophrenia and/or psychosis. There was one study that utilized healthy volunteers from a bioequivalence study of olanzapine [27]. Treatment length varied from a single dose to 7 years. In total, 19 studies utilized an untargeted approach to assess either metabolomic or lipidomic profiles or both. The primary tissue source for metabolomics was plasma (21 studies), followed by serum (15 studies). Three studies were conducted using brain tissue, which included the superior temporal gyrus (Atagun et al.), prefrontal cortex and hippocampus (Fuji et al.), and prefrontal cortex (Schwarz et al.) [10,19,39]. Antipsychotic medication varied widely across the studies, but detailed descriptions of current medication use were not always available. Medication and additional study characteristics are included in Supplementary Table S1. Study quality ranged from high to low and can be found in Supplementary Tables S6–S10.

### 3.2. Untargeted Metabolomic Studies

Fourteen studies used an untargeted metabolomic approach. Reporting of the full metabolomic data set available for analysis (e.g., total ions identified, known metabolites identified, etc.) was mixed. Additionally, analysis strategies varied across the studies and included multivariate (e.g., partial least squares-discriminant analysis, etc.) and univariate approaches (e.g., *t*-tests with multiple testing corrections). Seven studies found > 10 significant metabolites associated with antipsychotic use, while six studies found < 10 metabolites, and one study found no associations [22] Fifty-four associations with metabolite classes were identified across all studies, with carboxylic acids being the most commonly identified in nine studies. General metabolite class identifications are detailed in Table 2, and in-depth findings are provided in Supplementary Table S2.

### 3.3. Untargeted Lipidomic Studies

Eight studies used an untargeted lipidomic approach to explore lipid metabolite associations with antipsychotic treatment. These studies involved analyzing anywhere from several hundred to more than a thousand lipid metabolites in a sample and evaluated a total of 11 lipid classes. Numerous significant lipid metabolites associated with antipsychotic treatment were identified in every study, with 29 total lipid class associations across all studies. The most commonly associated lipid class with antipsychotic treatment was phosphatidylcholines (five studies). Three studies included analyses of metabolites associated with antipsychotic response [9,17,42]. A summary of the untargeted lipidomic studies is in Table 3 and Supplementary Table S3.

### 3.4. Targeted Metabolomic Studies

Fifteen studies used targeted approaches to investigate metabolomic changes with antipsychotic use (Table 4 and Supplementary Table S4). Targeted metabolite classes included neurotransmitters, sugars, tryptophan metabolites, amino acids, acylcarnitines, cytokines, growth factors, amines, amides, organic acids, oxidative metabolites, endocanabinoids, eicosanoids, purines, or a combination of these metabolite classes with data on select lipids [20,23,24,31,32]. A total of 803 metabolites were analyzed in all studies, with 91 significant associations identified. Seven metabolites showed replicated associations (aspartate, 5-hydroxyindoleadceitc acid, glutamine, kynurenine, oleylcarnitine, stearoylcarnitine, and tiglylcarnitine). The five studies that measured lipid and non-lipid metabolites were included in this section of the review. Three studies utilized the commercially available Biocrates AbosluteIDQ^®^ metabolomic kits [20,23,36]. 

### 3.5. Targeted Lipidomic Studies

Ten studies investigated lipidomic changes utilizing a targeted approach (Table 5 and Supplementary Table S5). The number of targeted lipids ranged from 4 to greater than 290 per study, with a total of 747 lipid metabolites analyzed across all studies. Cumulatively, lipids that increased in concentration upon antipsychotic exposure included ceramides, fatty acids, polyunsaturated fatty acids, and phosphatidylcholines. Decreases in lipids were observed in four studies, including in phosphatidylcholines, phosphatidylserines, sphingomyelins, and fatty acids. Three lipid species were found to be replicated and included eicosapentaenoic acid, FA22.46, and PC40:6. Only one study identified no significant lipid changes with antipsychotic treatment [12].

## 4. Discussion

**Figure 1:**
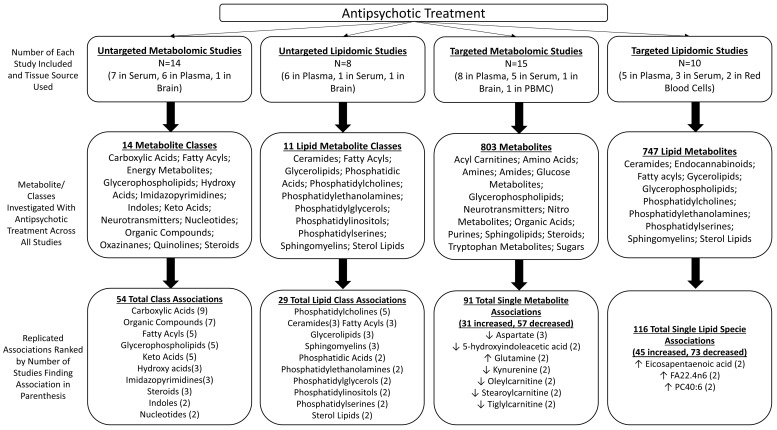
Summarized Findings. The figure outlines the findings of studies included in this review that have investigated the effects of antipsychotics on the metabolome and lipidome through both untargeted and targeted studies. The first row of bubbles indicates the number of included studies for the four categories of untargeted metabolomic studies, untargeted lipidomic studies, targeted metabolic studies, and targeted lipidomic studies. Within these bubbles, the type of tissue or medium used for the omic analysis is indicated. Of note, one study in the untargeted metabolomic and lipidomic categories (each) used multiple tissue sources, two studies in the targeted metabolomic category, and three studies in the targeted lipidomics category (see tables for details). The second row of bubbles gives the total number of metabolite/lipid classes analyzed for untargeted studies and the total number of individual metabolites/lipids analyzed for targeted studies, along with their classes. For example, in the left, second-row bubble, the 14 included untargeted metabolomic studies analyzed a total of 14 metabolite classes, which are listed in the bubble. The final third row of bubbles gives the summarized findings for the included studies. Each bubble gives a broad overview of the findings of all studies. For example, 54 total metabolite class associations were identified across all the untargeted metabolomic studies. Each bubble also gives the metabolite/lipid classes or individual metabolite/lipids with replicated associations in the included studies. The number of replications is indicated in parentheses and for the targeted studies, the directionality of the replications are indicated by an up (increased) or down (decreased) arrow.

This systematic review summarized the literature regarding antipsychotic effects on metabolomic and lipidomic profiles in humans, as assessed with mass spectrometry. To that end, 42 studies were identified that met the inclusion criteria and were published from 2007 to 2022. The included studies were quite varied in their design and approach to assessing metabolites (i.e., targeted versus untargeted); however, 88% of the studies were conducted in patients with psychosis, and 50% analyzed metabolite profiles in the plasma. Furthermore, 80% of studies reported assessing the metabolomic associations with atypical antipsychotics. Although highly heterogeneous, some commonalities and findings among the studies can be discussed (Figure 1).

### 4.1. Findings in Untargeted Metabolomic Studies

Amongst the 14 untargeted metabolomic studies, the most frequently identified class of metabolites associated with antipsychotic treatment was carboxylic acids (nine studies). The carboxylic acid class of metabolites is large and contains many compounds that have the carboxylic acid moiety attached [52]. It includes several critical groups of molecules such as amino acids, citric acid cycle metabolites, and fatty acids of various chain lengths. Amino acids have been suggested to play a role in both the pathophysiology of schizophrenia and response to antipsychotic treatment, likely due to their role as building blocks for neurotransmitters or functionality as neurotransmitters themselves [53,54]. Furthermore, genetic models of altered binding efficiency involving amino acids have been associated with schizophrenia symptoms [55]. This research and possibly findings from the studies included here have led to investigations into the utilization of amino acids and related compounds in the treatment of schizophrenia [56]. 

The next most common metabolite class associated with antipsychotic treatment amongst the untargeted metabolomic studies were the keto acids and organic compounds. Keto acids consist of compounds with a ketone and carboxylic acid group and can be influenced both by medication and dietary interventions [57,58]. The metabolism of ketones has been linked to cardiovascular disease, which may be relevant to the cardiovascular disease associated with antipsychotic treatment [59,60]. Indeed, one study investigated the effect of ketogenic diets in animal models treated with antipsychotics and found that a diet-based approach to increasing ketones may be helpful in reducing antipsychotic-associated hyperglycemia [61]. Limited case studies in humans have also suggested that a ketogenic diet may be helpful in the management of schizophrenia symptoms and metabolic disease [62]. The other common group, organic compounds, primarily consisted of organooxygen compounds (e.g., glucose, glucuronic acid, etc.), organic sulfonic acids (e.g., taurine, etc.), and organonitrogen compounds (e.g., Trimethylamine N-oxide (TMAO), etc.). Beyond the well-known effects of antipsychotics on glucose metabolism, one study found that adjunctive taurine treatment in patients with first-episode psychosis had improved symptoms of psychosis, as measured by the Brief Psychiatric Rating Scale [63]. Furthermore, organonitrogen compounds like TMAO have been linked to general cardiovascular disease and microbiome (brain–gut) health [64,65]. Together, this suggests a potential role for organonitrogen compounds like that of TMAO in antipsychotic efficacy and side effects.

### 4.2. Findings in Untargeted Lipidomic Studies

Within the untargeted lipidomic studies, the most common lipid classes associated with antipsychotic treatment included phosphatidylcholines (5 studies), triglycerides (4 studies), phosphatidylethanolamines (3 studies), sphingomyelins (3 studies), ceramides (2 studies), and diacylglycerides (2 studies). Alterations in lipid profiles have long been associated with antipsychotic treatment; therefore, advanced profiling of lipid species with lipidomics has been a natural progression from that of a simple, clinical lipid profile. Changes in lipid profiles have not only been cited as a side effect but also an indicator of antipsychotic efficacy, and it has been hypothesized that changes in lipid metabolism are among possible pathogenic mechanisms of schizophrenia [66,67]. Yet, the mechanism by which antipsychotics induce lipid alterations is still debated and includes influencing lipid biosynthesis, metabolism, trafficking, and signaling, in addition to altered gene activity [68,69,70,71]. The findings in the untargeted studies included here suggest that antipsychotics have widespread effects on various lipid classes within the body. Interestingly, there is also work indicating that lipid-lowering therapy in antipsychotic-treated patients should be considered in study design when utilizing lipidomics to elucidate mechanisms of mental illness or cardiovascular side effects of antipsychotics [72].

### 4.3. Targeted Metabolomic and Lipidomic Findings

For the targeted metabolomic studies, multiple studies identified changes in acylcarnitines as being significantly associated with antipsychotic use. The acylcarnitines are a class of fatty acyls that have wide-reaching effects, including roles in energy production, energy homeostasis, lipid metabolism, inflammation, and insulin sensitivity [73,74,75,76,77], but their hypothesized associations with antipsychotic treatment seem apparent. Future work will need to better define which acylcarnitines are predictive of treatment outcomes with antipsychotics, whether genetics of acylcarnitines metabolism plays a role in antipsychotic outcomes, and if carnitine-based therapies, which to date have mixed findings, have a place in antipsychotic treatment [78,79,80,81]. 

Two studies found significant alterations in neurotransmitters [14,27], while one study found no effect [10], which is notable as both of the studies found significant associations utilizing peripheral sources (i.e., plasma) for their investigations, while the study with no associations utilized brain samples. This could suggest that circulating neurotransmitters may have biomarker potential for psychopharmacologic treatment. In contrast to some of the positive findings described above, both studies on tryptophan metabolites and one study on oxidative metabolites did not identify significant associations with antipsychotic treatment [16,29,49].

Three studies within this group utilized the commercially available and validated Absolute IDQ^®^ kits [20,23,36]. The use of such a kit is important as it begins to create a standardized metabolomic workflow that allows for comparisons between studies and can be applied clinically. With the three studies that utilized the AbosoluteIDQ platform, the findings were heterogeneous; however, this is partly because investigators performed sub-analyses on certain classes of metabolites measured by the platform while excluding other classes from the analysis. For example, He and colleagues utilized all metabolites measured by the P150 kit and found only one statistically significant change in phosphatidylcholine that remained after correction for multiple tests [20]. The second study by Krissa and colleagues only analyzed acylcarnitines from the kit but found several significant associations with antipsychotic use [23]. Finally, Parksepp only analyzed amino acids and biogenic amines from the kit but found several significant associations with antipsychotics [36]. Going forward, these commercially available metabolomic kits, coupled with fully reported data that includes non-significant metabolites, will enable approaches such as meta-analysis to better understand those metabolites that are truly associated with antipsychotic treatment.

The targeted lipidomic studies, of which there were 10, showed similar results to the untargeted studies in that there were changes identified in many of the untargeted lipid classes described above (e.g., phosphatidylcholines, fatty acyls, etc.). Eight of the ten studies found an increase in at least one lipid, one study only found decreases in lipids, and one study found no changes with antipsychotic treatment. One novel lipid class analyzed in the targeted studies is the endocannabinoids. The endocanabinoids are derived from polyunsaturated fatty acids and have roles in lipid signaling which, in turn, has broad potential effects on processes ranging from inflammation to cognition [82]. The endocanbinoid system is highly complex in its composition, regulation, and interactions and has been studied in the pathogenesis of schizophrenia and bipolar disorder [83,84,85]. Further studies targeting this system will reveal further information on its role in antipsychotic treatment.

### 4.4. Limitations of the Review and Current Literature

One limitation of the current review and literature is the lack of a quantitative synthesis (i.e., a meta-analysis of the results). The primary reason for this is due to the non-complete results presented in the included studies. In most instances, studies present only those metabolites (or lipids) that are statistically significant, and this is especially true for untargeted approaches or approaches that use multivariate statistical methods. Going forward, either data transparency or presentation of all metabolite statistics, regardless of significance, should be presented in supplementary files or uploaded to repositories such as the Metabolomics Workbench. This will allow for meta-analytic approaches that will not be biased to only capture significant associations that are generally published. Despite this limitation, the current review was thorough and extensively summarized 42 studies. Another limitation of the current literature is the heterogeneous study type, antipsychotic type, and metabolomics approach. Nevertheless, the summary provided above aims to provide future metabolomics research with a foundation for the design of experiments based on past findings. Another limitation of this review is that it covers many metabolite and lipid classes, which is potentially not useful to those studying or treating mental illness holistically and not at the molecular level. We hope that one may identify a subset of studies or metabolite classes of interest that will allow the reader to go more fully in-depth using other reviews or the studies themselves. Finally, the quality of the included studies was mixed, and only one study was a randomized controlled trial of high quality. To strengthen associations, future study designs should rely on causal designs.

## 5. Conclusions

The current literature regarding the metabolomic and lipidomic changes associated with antipsychotic use is highly varied and complex. Nevertheless, certain metabolites and lipids such as neurotransmitters, carboxylic acids, and acylcarnitines have garnered repeated associations across studies, yet, at this time, no single metabolite or lipid has shown enough evidence for application clinically. Going forward, it will be critical that metabolomic and lipidomic studies carefully design their studies to achieve causality and fully report all metabolite or lipid associations, including non-significant statistics, thus allowing quantitative synthesis and more objective summarizations of the field. Further investigations will allow for panel development that could lead to biomarker utility with antipsychotic treatment or the development of novel therapeutics that can be coupled with antipsychotic treatment.

## Figures and Tables

**Table 1:** Characteristics of Included Studies.

Study	Tissue	Study Design	Participant Diagnoses	Treatment Length	Type of Study
Al Awam 2015 [8]	P	CC	26 Scz	NR	UM, UL
Aquino 2018 [9]	P	PP	54 Scz	6 weeks	UL
Atagün 2018 [10]	B ^b^	CC	29 BP	NR	TM
Bicikova 2013 [11]	S	PP ^a^	21 Scz	6 months	TM
Brunkhorst-Kanaan 2019 [12]	P	CC	67 MDD/BP	NR	TL
Buretić-Tomljanović 2008 [13]	S	PP ^a^	44 Scz	7 months	TM
Cai 2012 [14]	P/U	PP ^a^	11 Scz	6 weeks	UM, TM
Cao 2019 [15]	S	PP	122 Scz	8 weeks	UM
Condray 2011 [16]	P	PP^a^	24 Scz	4 weeks	TM
deAlmeida 2020 [17]	P	PP	54 Scz	6 weeks	UL
Evans 2014 [18]	P	CC	54 BP	NA	TL
Fujii 2017 [19]	B ^b^	CC	29 Scz	NA	UM
He 2012 [20]	P	CC	265 Scz	NA	TM
Kaddurah-Daouk 2007 [21]	P	PP ^a^	50 Scz	2–3 weeks	TL
Kim 2022 [22]	S	PP ^a^	40 Scz	NR	UM
Kriisa 2017 [23]	S	PP ^a^	36 Scz	7 months	TM
Lenski 2021 [24]	P	PP	86 Scz	1 month	TM
Li 2022 [25]	RBC	PP ^a^	327 Scz	4 weeks	TL
Liu 2015 [26]	PBMC	CS	20 Scz	NA	TM
Liu 2020 [27]	P	PP ^a^	17 HC	single dose ^b^	UM, TM
Liu 2021 [28]	P	PP	27 Scz	4 weeks	UM
Maes 2019 [29]	P	CS	37 MDD, 45 BPI, 23 BPII	NA	TM
McEvoy 2013 [30]	P	RCT ^a^	40 Scz	2 weeks	TL
Mednova 2021 [31]	S	CS	37 Scz	NR	TM
Mednova 2022 [32]	S	CS	112 Scz	7 years	TM
Okamoto 2021 [33]	S	CC	30 Scz	NR	UM
Orešič 2011 [34]	S	CS	19 Scz, 57 ONAP, 37 AfPs	NA	UM
Paredes 2014 [35]	P	CS	60 Scz	NA	UM, UL
Parksepp 2020 [36]	S	PP ^a^	52 Scz	6 months and 5 years	TM
Parksepp 2022 [37]	S	PP ^a^	112 Scz	6 months and 5 years	TL
Qiao 2016 [38]	S	PP ^a^	15 Scz	4 weeks	UM
Schwarz 2008 [39]	B ^b^, RBC	CS	35 Scz15 BP	NA	UL
Suvitaival 2016 [40]	S	PP	36 Scz	2 months, 1 year	UM, UL
Tessier 2016 [41]	RBC	CS	74 Scz	NA	TL
Tkachev 2021 [42]	P	PP	92 Scz	37 days	UL
Wang 2018 [43]	S	PP ^a^	115 Scz	8 weeks	TL
Wang 2022 [44]	P	PP	25 Scz	4 weeks	UM
Wood 2015 [45]	P, PLT	CS	23 Scz	NA	TL
Xuan 2011 [46]	S	PP ^a^	18 Scz	8 weeks	UM
Yan 2018 [47]	P	PP ^a^	20 Scz	8 weeks	UL
Yao 2010a [48]	P	PP ^a^	25 Scz	4 weeks	TM
Yao 2010b [49]	P	PP ^a^	25 Scz	4 weeks	TM

^a^ A healthy control group was included with the pre-post design to compare to the baseline sample prior to treatment. This is not reported in this systematic review since we are concerned with treatment effects. ^b^ The brain regions investigated were as follows: Atagun et al. superior temporal gyrus; Fuji et al. prefrontal cortex and hippocampus Schwarz et al. prefrontal cortex [10,19,39]. Abbreviations: AfPs = affective psychosis, B = brain, BP = bipolar disorder, CC = case-control, CS = cross-sectional, HC = health control, MDD = major depressive disorder, NA = Not applicable, NR = not reported, ONAP = other non-affective psychosis, P = plasma, PBMC = peripheral blood mononuclear cells, PLT = platelets, PP = pre-post, RBC = red blood cells, RCT = randomized controlled trial, S = serum, SCZ = schizophrenia, TL = targeted lipidomics, TM = targeted metabolomics, U = urine, UL = untargeted lipidomics, UM = untargeted metabolomics.

**Table 2:** Metabolite Class Changes with Antipsychotic Treatment—Results from Untargeted Metabolomic Studies. Markings indicate metabolite class(es) with statistically significant associations with antipsychotic treatment.

Study	Tissue	CA	FA	E	GPL	HA	Imid	Ind	Ka	Nt	Nuc	Oc	Ox	Q	S
Al Awam 2015 [8]	P	x	x				x					x			x
Cai 2012 [14]	P/U	x			x	x			x			x			x
Cao 2019 [15]	S	x			x				x						
Fujii 2017 [19]	B	x	x				x						x		
Kim 2022 [22]	S														
Liu 2020 [27]	P	x			x					x	x				
Liu 2021 [28]	P				x							x			
Okamoto 2021 [33]	S	x						x			x	x			
Orešič 2011 [34]	S		x	x					x			x			
Paredes 2014 [35]	P	x				x			x					x	
Qiao 2016 [38]	S	x			x				x			x			
Suvitaival 2016 [40]	S	x													
Wang 2022 [44]	P		x												
Xuan 2011 [46]	S		x			x	x	x				x			x

Abbreviations: B = brain; CA = carboxylic acids; FA = fatty acyl or lipid-like molecule; E = energy metabolites; GPL = glycerophospholipids; HA = hydroxy acids; Imid= imidazopyrimidines; Ind = indoles; Ka = keto acids; Nt = neurotransmitters; Nuc = nucleotides; Oc = organic compounds; Ox= oxazinanes; P = plasma; Q = quinolines; S = serum; S = steroids; U = urine.

**Table 3:** Lipid Metabolite Class Changes with Antipsychotic Treatment—Results from Untargeted Lipidomic Studies. Markings indicate metabolite class(es) with statistically significant associations with antipsychotic treatment.

Study	Tissue	Cer	FA	GL	PA	PC	PE	PG	PI	PS	SM	SL
Al Awam 2015 [8]	P		x									x
Aquino 2018 ^a^ [9]	P	x			x	x		x	x	x	x	
deAlmeida 2020 ^a^ [17]	P	x		x	x	x		x	x	x	x	
Paredes 2014 ^a^ [35]	P			x								
Schwarz 2008 [39]	B, RBC		x			x						
Suvitaival 2016 ^a^ [40]	S					x	x					
Tkachev 2021 [42]	P			x								
Yan 2018 [47]	P	x	x			x	x				x	x

^a^ changes dependent on individual antipsychotic medication or medication grouping (e.g., low versus high-risk for side effects) Abbreviations: B = brain; Cer = ceramide; FA = fatty acyl; GL = glycerolipid; PA = phosphatidic acids; PC = phosphatidylcholine; PE = phosphatidylethanolamine; PG = phosphatidylglycerol; PI = phosphatidylinositol; P = plasma; PS = phosphatidylserine; S = serum; SM = sphingomyelin; SL = sterol lipid; RBC = red blood cell.

**Table 4:** Overview of Metabolomic Associations with Antipsychotic Use in Targeted Studies. Arrows provide directional changes of significant associations.

Study	Tissue	# Metabolites	Metabolites Targeted	Significant Results
Atagün 2018 [10]	B	6	Neurotransmitters and sugars	No significant changes
Bicikova 2013 [11]	S	31	Steroids	↑ androsterone, ↓ 5,20-tetrahydroprogesterone, ↓ etiocholanolone, ↓ pregnenolone sulfate
Cai 2012 [14]	P/U	11	Neurotransmitters	Multivariate significance that included all metabolites. Shifts included ↑ glutamate, ↑ glutamine, ↓ dopamaine, ↑ dihydroxyphenylacetic acid, ↑ homovanillic acid, ↑ norepinephrine, ↑ vanillylmandelic acid, ↓ 3-methoxy-4-hydroxyphenylglycol, ↑ 5-hydroxytryptamine, ↓ 5-hydroxyindoleacetic acid
Condray 2011 [16]	P	7	Tryptophan metabolites	No significant changes
He 2012 [20]	P	163	AbsoluteIDQ p150^®^ kit ^ab^	PC acyl-akyl C34:3 ^e^
Kriisa 2017 [23]	S	206	AbsoluteIDQ p180^®^ kit ^bcd^	↓ C16 hexadecanoyl-carnitine, ↓ C18:1 octadecenoyl-carnitine, ↓ C18:2 octadecadienyl-carnitine, ↑ C3 propionyl-carnitine
Lenski 2021 [24]	P	220	Acylcarnitines, amino acids, amines, amides, tryptophan metabolites, organic acids, and sugars ^b^	Significant metabolites pre to post included 5 amino acids (1 ↑ and 4 ↓) ^g^, 6 acylcarnitines (5 ↑ and 1 ↓), 4 ↑ carboxylic acids, 1 ↓ catecholamine, 1 ↑ nucleoside, 1 ↓ pyridine, and 1 ↑ tetrapyrrole.
Liu 2015 [26]	PBMC	13	Glucose metabolism pathway	↑ Ribose 5-phosphate
Liu 2020 [27]	P	13	Neurotransmitters	↓ L-tryptophan, ↓ L-tyrosine, ↓ 5-hydroxytryptophan, ↓ 5-hydroxyindoleacetic acid, ↓ γ-aminobutyric acid, ↓ L-3,4-dihydroxyphenylalanine, ↓ taurine, ↓ kynurenine
Maes 2019 [29]	P	6	Nitro-oxidative and nitrosative stress pathways	No significant changes
Mednova 2021 [31]	S	45	Amino acids and acylcarnitines ^b^	↓ 8 amino acids and 12 acylcarnitines (1 ↑ and 11 ↓)
Mednova 2022 [32]	S	32	Amino acids and acylcarnitines ^b^	↑ alanine, ↓ C5, ↓ C5:1, ↓ C10, ↓ C10:1, ↓ C12, ↓ C18
Parksepp 2020 [36]	S	31	AbsoluteIDQ p180^® f^	↑ asparagine, ↓ aspartate, ↑ glutamine, ↓ glutamate, ↑ methionine, ↑ valine, ↓ α-amino-adipic acid, ↓ histamine, ↑ putrescine, ↑ taurine, ↓ α-amino-adipic acid/kynurenine ratio, ↓ aspartate/asparagine ratio, ↓ glutamate/glutamine ratio, ↑ ornithine/arginine ratio
Yao 2010a [48]	P	6	Purine pathway metabolites	↓ guanine and ↑ uric acid/guanosine ratio
Yao 2010b [49]	P	13	Tryptophan pathway metabolites	No significant changes

^a^ p150 kit includes acyl carnitines, amino acids, glycerophospholipids, sphingolipids, and hexose. ^b^ The targeted metabolic analysis also included lipid metabolites; ^c^ p180 kit includes classes in 150 kit plus biogenic amines and additional amino acids. The investigators only utilized acylcarnitine levels from the P180 kit. ^d^ This study included metabolomic changes measured by the Randox biochip which measured tumor necrosis factor-alpha (TNF-α), interferon-gamma (IFN-γ), interleukin 1 alpha (IL-1α), Interleukin 1 beta (IL-1β), Interleukin 2 (IL-2), Interleukin 4 (IL-4), Interleukin 6 (IL-6), Interleukin 8 (IL-8), Interleukin 10 (IL-10), Monocyte Chemoattractant Protein-1 (MCP-1), Vascular endothelial growth factor (VEGF), Epidermal growth factor (EGF), C-peptide, insulin leptin, resistin, ferritin, and Plasminogen activator inhibitor-1 (PAI-1). While the focus of this systematic review was MS-based metabolomic studies, the details of the metabolite changes measured by this biochip are in the supplementary material. ^e^ metabolite was significantly different pre- to post-treatment, but direction was not indicated. ^f^ This study did not analyze the lipid metabolite levels obtained by the AbsoluteIDQ^®^ kit but only the amino acid and biogenic amine levels. ^g^ The parenthetical increases (↑) and decreases (↓) refer to the number within that cited metabolite class; for example, five amino acids (1 ↑ and 4 ↓) means that one amino acid in that study increased with antipsychotic treatment while four decreased.

**Table 5:** Overview of Lipidomic Associations with Antipsychotic Use in Targeted Studies. Arrows provide directional changes of significant associations.

Study	Tissue	# Lipid Metabolites	Lipid Metabolites Targeted	Significant Results
Brunkhorst-Kanaan 2019 [12]	P	36	Cer, ECB, GPL, SM	No Significant Changes ^b^
Buretić-Tomljanović 2008 [13]	S	105	AbsoluteIDQ p150^®^ kit ^a^	↑ 2 LysoPC acyls, ↑ Total PC diacyls, ↑ 9 PCs, ↓ 2 SMs
Evans 2014 [18]	P	16	FA	↑ EPA, ↑ LA
Kaddurah-Daouk 2007 [21]	P	290	Cer, FA, GL, GPL, PC, PE	↓ PCs, ↓ Pes, ↓ DAGs, ↓ TGs, ↑ fatty acids
Li 2022 [25]	RBC	10	FA	↑ (all)
McEvoy 2013 [30]	P	43	GPL, PC, PE	↑ PE.n3, ↑ PE.n6
Parksepp 2022 [37]	S	55	ECB, GPL	↑ ECB (1 total), ↑ PC (4 total), ↓ PC (9 total)
Tessier 2016 [41]	RBC	128	PC, PE, PS, SM	↑ PS, ↓ SM
Wang 2018 [43]	S	49	FA	9 AA (5 ↑, 4 ↓) ^d^, 2 ↓ DHA, 2 ↓ EA, 1 ↓ EDA, 1 ↑ EPA, 7 ↓ LA
Wood 2015 [45]	P, PLT	15	FA, GPL	In the plasma, 5 ↓ PC, 4 ↓ PE, ↓ DHA ^c^

^a^ p150 kit includes acyl carnitines, amino acids, glycerophospholipids, sphingolipids, and hexose; however, the investigators limited their analysis to glycerophospholipids and sphingomyelins. ^b^ No significant effect of antipsychotics as a class but an effect of olanzapine in the sub-analysis. See supplementary information for details. ^c^ Study also analyzed platelets, which showed ↑ PC, ↓ PE, and ↓ DHA. ^d^ The parenthetical Increases (↑) and decreases (↓) refer to the number within that cited metabolite class; for example, 9 AA (5 ↑, 4 ↓) means that five arachidonic acid metabolites in that study increased with antipsychotic treatment while four decreased. Abbreviations: AA = arachidonic acid; Cer = ceramide; DHA = docosahexaenoic acid; EA = ethanolamide; ECB = endocannabinoid; EDA= eicosadienoic acid; EPA = eicosapentaenoic acid; FA = fatty acyl; GL = glycerolipid; GPL = glycerophospholipid; LA = γ-linoleic acid; PBMC = peripheral blood mononuclear cells, PC = phosphatidylcholine; PE = phosphatidylethanolamine; PLT = platelet; P = plasma, PS = phosphatidylserine; RBC = red blood cell; S = serum; SM = sphingomyelin.

## Data Availability

All data for this systematic review was extracted from published literature and, as such, can be found in the referenced studies.

## Supplementary Materials

The following supporting information can be downloaded at: https://www.mdpi.com/article/10.3390/biomedicines11123295/s1, Figure S1: Prisma Flow Diagram; Table S1. Included Study Antipsychotic Characteristics, Age, and Sex; Table S2. Detailed Findings of Untargeted Metabolomic Studies; Table S3. Detailed Findings of Untargeted Lipidomic Studies; Table S4. Detailed Findings of Targeted Metabolomic Studies; Table S5. Detailed Findings of Targeted Lipidomic Studies; Table S6. Quality Assessment of Cross-Sectional Studies using NHLBI Quality Assessment Tool; Table S7. Quality Assessment of Case-Control Studies using NHLBI Quality Assessment Tool; Table S8. Quality Assessment of Pre-Post Studies using NHLBI Quality Assessment Tool; Table S9. Quality Assessment of Pre-Post Studies using NHLBI Quality Assessment Tool; Table S10. Quality Assessment of Controlled Intervention Studies using NHLBI Quality Assessment Tool.

## References

[1] Radojčić M.R., Pierce M., Hope H., Senior M., Taxiarchi V.P., Trefan L., Swift E., Abel K.M. (2023). Trends in antipsychotic prescribing to children and adolescents in England: Cohort study using 2000-19 primary care data. Lancet Psychiatry.

[2] Luo H., Lau W.C.Y., Chai Y., Torre C.O., Howard R., Liu K.Y., Lin X., Yin C., Fortin S., Kern D.M. (2023). Rates of Antipsychotic Drug Prescribing Among People Living With Dementia During the COVID-19 Pandemic. JAMA Psychiatry.

[3] Shoham N., Cooper C., Lewis G., Bebbington P., McManus S. (2021). Temporal trends in psychotic symptoms: Repeated cross-sectional surveys of the population in England 2000-14. Schizophr. Res..

[4] Haddad P.M., Brain C., Scott J. (2014). Nonadherence with antipsychotic medication in schizophrenia: Challenges and management strategies. Patient Relat. Outcome Meas..

[5] Tiihonen J., Taipale H., Mehtälä J., Vattulainen P., Correll C.U., Tanskanen A. (2019). Association of Antipsychotic Polypharmacy vs Monotherapy With Psychiatric Rehospitalization Among Adults With Schizophrenia. JAMA Psychiatry.

[6] Page M.J., McKenzie J.E., Bossuyt P.M., Boutron I., Hoffmann T.C., Mulrow C.D., Shamseer L., Tetzlaff J.M., Akl E.A., Brennan S.E. (2021). The PRISMA 2020 statement: An updated guideline for reporting systematic reviews. BMJ.

[7] Emwas A.H. (2015). The strengths and weaknesses of NMR spectroscopy and mass spectrometry with particular focus on metabolomics research. Methods Mol. Biol..

[8] Al Awam K., Haußleiter I.S., Dudley E., Donev R., Brüne M., Juckel G., Thome J. (2015). Multiplatform metabolome and proteome profiling identifies serum metabolite and protein signatures as prospective biomarkers for schizophrenia. J. Neural Transm..

[9] Aquino A., Alexandrino G.L., Guest P.C., Augusto F., Gomes A.F., Murgu M., Steiner J., Martins-de-Souza D. (2018). Blood-Based Lipidomics Approach to Evaluate Biomarkers Associated With Response to Olanzapine, Risperidone, and Quetiapine Treatment in Schizophrenia Patients. Front. Psychiatry.

[10] Atagün M.İ., Şıkoğlu E.M., Can S.S., Uğurlu G.K., Kaymak S.U., Çayköylü A., Algın O., Phillips M.L., Moore C.M., Öngür D. (2018). Neurochemical differences between bipolar disorder type I and II in superior temporal cortices: A proton magnetic resonance spectroscopy study. J. Affect. Disord..

[11] Bicikova M., Hill M., Ripova D., Mohr P., Hampl R. (2013). Determination of steroid metabolome as a possible tool for laboratory diagnosis of schizophrenia. J. Steroid Biochem. Mol. Biol..

[12] Brunkhorst-Kanaan N., Klatt-Schreiner K., Hackel J., Schröter K., Trautmann S., Hahnefeld L., Wicker S., Reif A., Thomas D., Geisslinger G. (2019). Targeted lipidomics reveal derangement of ceramides in major depression and bipolar disorder. Metabolism.

[13] Buretić-Tomljanović A., Giacometti J., Nadalin S., Rubesa G., Vulin M., Tomljanović D. (2008). Phospholipid membrane abnormalities and reduced niacin skin flush response in schizophrenia. Psychiatr. Danub..

[14] Cai H.L., Li H.D., Yan X.Z., Sun B., Zhang Q., Yan M., Zhang W.Y., Jiang P., Zhu R.H., Liu Y.P. (2012). Metabolomic analysis of biochemical changes in the plasma and urine of first-episode neuroleptic-naïve schizophrenia patients after treatment with risperidone. J. Proteome Res..

[15] Cao B., Jin M., Brietzke E., McIntyre R.S., Wang D., Rosenblat J.D., Ragguett R.-M., Zhang C., Sun X., Rong C. (2019). Serum metabolic profiling using small molecular water-soluble metabolites in individuals with schizophrenia: A longitudinal study using a pre–post-treatment design. Psychiatry Clin. Neurosci..

[16] Condray R., Dougherty G.G., Keshavan M.S., Reddy R.D., Haas G.L., Montrose D.M., Matson W.R., McEvoy J., Kaddurah-Daouk R., Yao J.K. (2011). 3-Hydroxykynurenine and clinical symptoms in first-episode neuroleptic-naive patients with schizophrenia. Int. J. Neuropsychopharmacol..

[17] de Almeida V., Alexandrino G.L., Aquino A., Gomes A.F., Murgu M., Dobrowolny H., Guest P.C., Steiner J., Martins-de-Souza D. (2020). Changes in the blood plasma lipidome associated with effective or poor response to atypical antipsychotic treatments in schizophrenia patients. Prog. Neuro-Psychopharmacol. Biol. Psychiatry.

[18] Evans S.J., Ringrose R.N., Harrington G.J., Mancuso P., Burant C.F., McInnis M.G. (2014). Dietary intake and plasma metabolomic analysis of polyunsaturated fatty acids in bipolar subjects reveal dysregulation of linoleic acid metabolism. J. Psychiatr. Res..

[19] Fujii T., Hattori K., Miyakawa T., Ohashi Y., Sato H., Kunugi H. (2017). Metabolic profile alterations in the postmortem brains of patients with schizophrenia using capillary electrophoresis-mass spectrometry. Schizophr. Res..

[20] He Y., Yu Z., Giegling I., Xie L., Hartmann A.M., Prehn C., Adamski J., Kahn R., Li Y., Illig T. (2012). Schizophrenia shows a unique metabolomics signature in plasma. Transl. Psychiatry.

[21] Kaddurah-Daouk R., McEvoy J., Baillie R.A., Lee D., Yao J.K., Doraiswamy P.M., Krishnan K.R. (2007). Metabolomic mapping of atypical antipsychotic effects in schizophrenia. Mol. Psychiatry.

[22] Kim S., Okazaki S., Otsuka I., Shinko Y., Horai T., Shimmyo N., Hirata T., Yamaki N., Tanifuji T., Boku S. (2022). Searching for biomarkers in schizophrenia and psychosis: Case-control study using capillary electrophoresis and liquid chromatography time-of-flight mass spectrometry and systematic review for biofluid metabolites. Neuropsychopharmacol. Rep..

[23] Kriisa K., Leppik L., Balõtšev R., Ottas A., Soomets U., Koido K., Volke V., Innos J., Haring L., Vasar E. (2017). Profiling of Acylcarnitines in First Episode Psychosis before and after Antipsychotic Treatment. J. Proteome Res..

[24] Lenski M., Sidibé J., Gholam M., Hennart B., Dubath C., Augsburger M., von Gunten A., Conus P., Allorge D., Thomas A. (2021). Metabolomic alteration induced by psychotropic drugs: Short-term metabolite profile as a predictor of weight gain evolution. Clin. Transl. Sci..

[25] Li N., Yang P., Tang M., Liu Y., Guo W., Lang B., Wang J., Wu H., Tang H., Yu Y. (2022). Reduced erythrocyte membrane polyunsaturated fatty acid levels indicate diminished treatment response in patients with multi- versus first-episode schizophrenia. Schizophrenia.

[26] Liu M.L., Zhang X.T., Du X.Y., Fang Z., Liu Z., Xu Y., Zheng P., Xu X.J., Cheng P.F., Huang T. (2015). Severe disturbance of glucose metabolism in peripheral blood mononuclear cells of schizophrenia patients: A targeted metabolomic study. J. Transl. Med..

[27] Liu D., An Z.L., Li P.F., Chen Y.H., Zhang R.P., Liu L.H., He J.M., Abliz Z. (2020). A targeted neurotransmitter quantification and nontargeted metabolic profiling method for pharmacometabolomics analysis of olanzapine by using UPLC-HRMS. RSC Adv..

[28] Liu J.H., Chen N., Guo Y.H., Guan X.N., Wang J., Wang D., Xiu M.H. (2021). Metabolomics-based understanding of the olanzapine-induced weight gain in female first-episode drug-naïve patients with schizophrenia. J. Psychiatr. Res..

[29] Maes M., Landucci Bonifacio K., Morelli N.R., Vargas H.O., Barbosa D.S., Carvalho A.F., Nunes S.O.V. (2019). Major Differences in Neurooxidative and Neuronitrosative Stress Pathways Between Major Depressive Disorder and Types I and II Bipolar Disorder. Mol. Neurobiol..

[30] McEvoy J., Baillie R.A., Zhu H., Buckley P., Keshavan M.S., Nasrallah H.A., Dougherty G.G., Yao J.K., Kaddurah-Daouk R. (2013). Lipidomics reveals early metabolic changes in subjects with schizophrenia: Effects of atypical antipsychotics. PLoS ONE.

[31] Mednova I.A., Chernonosov A.A., Kasakin M.F., Kornetova E.G., Semke A.V., Bokhan N.A., Koval V.V., Ivanova S.A. (2021). Amino Acid and Acylcarnitine Levels in Chronic Patients with Schizophrenia: A Preliminary Study. Metabolites.

[32] Mednova I.A., Chernonosov A.A., Kornetova E.G., Semke A.V., Bokhan N.A., Koval V.V., Ivanova S.A. (2022). Levels of Acylcarnitines and Branched-Chain Amino Acids in Antipsychotic-Treated Patients with Paranoid Schizophrenia with Metabolic Syndrome. Metabolites.

[33] Okamoto N., Ikenouchi A., Watanabe K., Igata R., Fujii R., Yoshimura R. (2021). A Metabolomics Study of Serum in Hospitalized Patients With Chronic Schizophrenia. Front. Psychiatry.

[34] Orešič M., Tang J., Seppänen-Laakso T., Mattila I., Saarni S.E., Saarni S.I., Lönnqvist J., Sysi-Aho M., Hyötyläinen T., Perälä J. (2011). Metabolome in schizophrenia and other psychotic disorders: A general population-based study. Genome Med..

[35] Paredes R.M., Quinones M., Marballi K., Gao X., Valdez C., Ahuja S.S., Velligan D., Walss-Bass C. (2014). Metabolomic profiling of schizophrenia patients at risk for metabolic syndrome. Int. J. Neuropsychopharmacol..

[36] Parksepp M., Leppik L., Koch K., Uppin K., Kangro R., Haring L., Vasar E., Zilmer M. (2020). Metabolomics approach revealed robust changes in amino acid and biogenic amine signatures in patients with schizophrenia in the early course of the disease. Sci. Rep..

[37] Parksepp M., Haring L., Kilk K., Koch K., Uppin K., Kangro R., Zilmer M., Vasar E. (2022). The Expanded Endocannabinoid System Contributes to Metabolic and Body Mass Shifts in First-Episode Schizophrenia: A 5-Year Follow-Up Study. Biomedicines.

[38] Qiao Y., Zhang L., He S., Wen H., Yu Y.M., Cao C.H., Li H.F. (2016). Plasma metabonomics study of first-Episode schizophrenia treated with olanzapine in female patients. Neurosci. Lett..

[39] Schwarz E., Prabakaran S., Whitfield P., Major H., Leweke F.M., Koethe D., McKenna P., Bahn S. (2008). High Throughput Lipidomic Profiling of Schizophrenia and Bipolar Disorder Brain Tissue Reveals Alterations of Free Fatty Acids, Phosphatidylcholines, and Ceramides. J. Proteome Res..

[40] Suvitaival T., Mantere O., Kieseppä T., Mattila I., Pöhö P., Hyötyläinen T., Suvisaari J., Orešič M. (2016). Serum metabolite profile associates with the development of metabolic co-morbidities in first-episode psychosis. Transl. Psychiatry.

[41] Tessier C., Sweers K., Frajerman A., Bergaoui H., Ferreri F., Delva C., Lapidus N., Lamaziere A., Roiser J.P., De Hert M. (2016). Membrane lipidomics in schizophrenia patients: A correlational study with clinical and cognitive manifestations. Transl. Psychiatry.

[42] Tkachev A., Stekolshchikova E., Anikanov N., Zozulya S., Barkhatova A., Klyushnik T., Petrova D. (2021). Shorter chain triglycerides are negatively associated with symptom improvement in schizophrenia. Biomolecules.

[43] Wang D., Sun X., Yan J., Ren B., Cao B., Lu Q., Liu Y., Zeng J., Huang N., Xie Q. (2018). Alterations of eicosanoids and related mediators in patients with schizophrenia. J. Psychiatr. Res..

[44] Wang X., Xiu M., Wang K., Su X., Li X., Wu F. (2022). Plasma linoelaidyl carnitine levels positively correlated with symptom improvement in olanzapine-treated first-episode drug-naïve schizophrenia. Metabolomics.

[45] Wood P.L., Unfried G., Whitehead W., Phillipps A., Wood J.A. (2015). Dysfunctional plasmalogen dynamics in the plasma and platelets of patients with schizophrenia. Schizophr. Res..

[46] Xuan J., Pan G., Qiu Y., Yang L., Su M., Liu Y., Chen J., Feng G., Fang Y., Jia W. (2011). Metabolomic Profiling to Identify Potential Serum Biomarkers for Schizophrenia and Risperidone Action. J. Proteome Res..

[47] Yan L., Zhou J., Wang D., Si D., Liu Y., Zhong L., Yin Y. (2018). Unbiased lipidomic profiling reveals metabolomic changes during the onset and antipsychotics treatment of schizophrenia disease. Metabolomics.

[48] Yao J.K., Dougherty G.G., Reddy R.D., Keshavan M.S., Montrose D.M., Matson W.R., McEvoy J., Kaddurah-Daouk R. (2010). Homeostatic Imbalance of Purine Catabolism in First-Episode Neuroleptic-Naive Patients with Schizophrenia. PLoS ONE.

[49] Yao J.K., Dougherty G.G., Reddy R.D., Keshavan M.S., Montrose D.M., Matson W.R., Rozen S., Krishnan R.R., McEvoy J., Kaddurah-Daouk R. (2010). Altered interactions of tryptophan metabolites in first-episode neuroleptic-naive patients with schizophrenia. Mol. Psychiatry.

[50] Kageyama Y., Kasahara T., Morishita H., Mataga N., Deguchi Y., Tani M., Kuroda K., Hattori K., Yoshida S., Inoue K. (2017). Search for plasma biomarkers in drug-free patients with bipolar disorder and schizophrenia using metabolome analysis. Psychiatry Clin. Neurosci..

[51] Ward K.M., Yeoman L., McHugh C., Kraal A.Z., Flowers S.A., Rothberg A.E., Karnovsky A., Das A.K., Ellingrod V.L., Stringer K.A. (2018). Atypical Antipsychotic Exposure May Not Differentiate Metabolic Phenotypes of Patients with Schizophrenia. Pharmacotherapy.

[52] Guo K., Li L. (2010). High-performance isotope labeling for profiling carboxylic acid-containing metabolites in biofluids by mass spectrometry. Anal. Chem..

[53] De Luca V., Viggiano E., Messina G., Viggiano A., Borlido C., Viggiano A., Monda M. (2008). Peripheral amino Acid levels in schizophrenia and antipsychotic treatment. Psychiatry Investig..

[54] Saleem S., Shaukat F., Gul A., Arooj M., Malik A. (2017). Potential role of amino acids in pathogenesis of schizophrenia. Int. J. Health Sci..

[55] Labrie V., Lipina T., Roder J.C. (2008). Mice with reduced NMDA receptor glycine affinity model some of the negative and cognitive symptoms of schizophrenia. Psychopharmacology.

[56] de Bartolomeis A., Vellucci L., Austin M.C., De Simone G., Barone A. (2022). Rational and Translational Implications of D-Amino Acids for Treatment-Resistant Schizophrenia: From Neurobiology to the Clinics. Biomolecules.

[57] Effinger D., Hirschberger S., Yoncheva P., Schmid A., Heine T., Newels P., Schütz B., Meng C., Gigl M., Kleigrewe K. (2023). A ketogenic diet substantially reshapes the human metabolome. Clin. Nutr..

[58] Yurista S.R., Chong C.R., Badimon J.J., Kelly D.P., de Boer R.A., Westenbrink B.D. (2021). Therapeutic Potential of Ketone Bodies for Patients With Cardiovascular Disease: JACC State-of-the-Art Review. J. Am. Coll. Cardiol..

[59] Cotter D.G., Schugar R.C., Crawford P.A. (2013). Ketone body metabolism and cardiovascular disease. Am. J. Physiol. Heart Circ. Physiol..

[60] Lai F.T.T., Guthrie B., Mercer S.W., Smith D.J., Yip B.H.K., Chung G.K.K., Lee K.P., Chung R.Y., Chau P.Y.K., Wong E.L.Y. (2020). Association between antipsychotic use and acute ischemic heart disease in women but not in men: A retrospective cohort study of over one million primary care patients. BMC Med..

[61] Shamshoum H., Medak K.D., McKie G.L., Hahn M.K., Wright D.C. (2022). Fasting or the short-term consumption of a ketogenic diet protects against antipsychotic-induced hyperglycaemia in mice. J. Physiol..

[62] Sarnyai Z., Kraeuter A.K., Palmer C.M. (2019). Ketogenic diet for schizophrenia: Clinical implication. Curr. Opin. Psychiatry.

[63] O'Donnell C.P., Allott K.A., Murphy B.P., Yuen H.P., Proffitt T.M., Papas A., Moral J., Pham T., O'Regan M.K., Phassouliotis C. (2016). Adjunctive Taurine in First-Episode Psychosis: A Phase 2, Double-Blind, Randomized, Placebo-Controlled Study. J. Clin. Psychiatry.

[64] Canyelles M., Borràs C., Rotllan N., Tondo M., Escolà-Gil J.C., Blanco-Vaca F. (2023). Gut Microbiota-Derived TMAO: A Causal Factor Promoting Atherosclerotic Cardiovascular Disease?. Int. J. Mol. Sci..

[65] Hoyles L., Pontifex M.G., Rodriguez-Ramiro I., Anis-Alavi M.A., Jelane K.S., Snelling T., Solito E., Fonseca S., Carvalho A.L., Carding S.R. (2021). Regulation of blood-brain barrier integrity by microbiome-associated methylamines and cognition by trimethylamine N-oxide. Microbiome.

[66] Kim D.D., Barr A.M., Fredrikson D.H., Honer W.G., Procyshyn R.M. (2019). Association between Serum Lipids and Antipsychotic Response in Schizophrenia. Curr. Neuropharmacol..

[67] Horrobin D.F., Glen A.I., Vaddadi K. (1994). The membrane hypothesis of schizophrenia. Schizophr. Res..

[68] Skrede S., Steen V.M., Fernø J. (2013). Antipsychotic-induced increase in lipid biosynthesis: Activation through inhibition?. J. Lipid Res..

[69] Pereira S., Au E., Agarwal S.M., Wright D.C., Hahn M.K. (2023). Antipsychotic-Induced Alterations in Lipid Turnover. Endocrinology.

[70] Vantaggiato C., Panzeri E., Citterio A., Orso G., Pozzi M. (2019). Antipsychotics Promote Metabolic Disorders Disrupting Cellular Lipid Metabolism and Trafficking. Trends Endocrinol. Metab..

[71] Cai H.L., Tan Q.Y., Jiang P., Dang R.L., Xue Y., Tang M.M., Xu P., Deng Y., Li H.D., Yao J.K. (2015). A potential mechanism underlying atypical antipsychotics-induced lipid disturbances. Transl. Psychiatry.

[72] Kanagasundaram P., Lee J., Prasad F., Costa-Dookhan K.A., Hamel L., Gordon M., Remington G., Hahn M.K., Agarwal S.M. (2021). Pharmacological Interventions to Treat Antipsychotic-Induced Dyslipidemia in Schizophrenia Patients: A Systematic Review and Meta Analysis. Front. Psychiatry.

[73] Schooneman M.G., Vaz F.M., Houten S.M., Soeters M.R. (2013). Acylcarnitines: Reflecting or inflicting insulin resistance?. Diabetes.

[74] McCoin C.S., Knotts T.A., Adams S.H. (2015). Acylcarnitines—Old actors auditioning for new roles in metabolic physiology. Nat. Rev. Endocrinol..

[75] Dambrova M., Makrecka-Kuka M., Kuka J., Vilskersts R., Nordberg D., Attwood M.M., Smesny S., Sen Z.D., Guo A.C., Oler E. (2022). Acylcarnitines: Nomenclature, Biomarkers, Therapeutic Potential, Drug Targets, and Clinical Trials. Pharmacol. Rev..

[76] Li S., Gao D., Jiang Y. (2019). Function, Detection and Alteration of Acylcarnitine Metabolism in Hepatocellular Carcinoma. Metabolites.

[77] Rutkowsky J.M., Knotts T.A., Ono-Moore K.D., McCoin C.S., Huang S., Schneider D., Singh S., Adams S.H., Hwang D.H. (2014). Acylcarnitines activate proinflammatory signaling pathways. Am. J. Physiol. Endocrinol. Metab..

[78] Nakamura M., Nagamine T. (2017). Pituitary gamma-aminobutyric acid receptor stimulation by carnitine may be a new strategy for antipsychotic-induced hyperprolactinemia. Psychiatry Clin. Neurosci..

[79] Evcimen H., Mania I., Mathews M., Basil B. (2007). Psychosis Precipitated by Acetyl-l-Carnitine in a Patient With Bipolar Disorder. Prim. Care Companion J. Clin. Psychiatry.

[80] Kulkarni S., Biswal J., Mohapatra D., Mishra S.N., Sahoo S. (2022). Acetyl-L-Carnitine as An Add-On Therapy for Negative and Cognitive Symptoms of Schizophrenia: A Randomized Control Trial. Indian J. Psychiatry.

[81] Bruno A., Pandolfo G., Crucitti M., Lorusso S., Zoccali R.A., Muscatello M.R. (2016). Acetyl-L-Carnitine Augmentation of Clozapine in Partial-Responder Schizophrenia: A 12-Week, Open-Label Uncontrolled Preliminary Study. Clin. Neuropharmacol..

[82] Astarita G., Piomelli D. (2009). Lipidomic analysis of endocannabinoid metabolism in biological samples. J. Chromatogr. B Analyt. Technol. Biomed. Life Sci..

[83] Seabra G., Falvella A.C.B., Guest P.C., Martins-de-Souza D., de Almeida V. (2018). Proteomics and Lipidomics in the Elucidation of Endocannabinoid Signaling in Healthy and Schizophrenia Brains. Proteomics.

[84] Desfossés J., Stip E., Bentaleb L.A., Potvin S. (2010). Endocannabinoids and Schizophrenia. Pharmaceuticals.

[85] Garani R., Watts J.J., Mizrahi R. (2021). Endocannabinoid system in psychotic and mood disorders, a review of human studies. Prog. Neuropsychopharmacol. Biol. Psychiatry.

