# Supplementary material for: Metabolomics, Lipidomics, and Antipsychotics: A Systematic Review
Source: Biomedicines. 2023 Dec 13;11(12):3295. doi: 10.3390/biomedicines11123295 (PMC10741000; doi:10.3390/biomedicines11123295)
Supplement: Supplementary file 1 [file biomedicines-11-03295-s001.zip › biomedicines-2721379-supplementary.pdf]

Supplementary Information

Figure S1. Prisma Flow Diagram

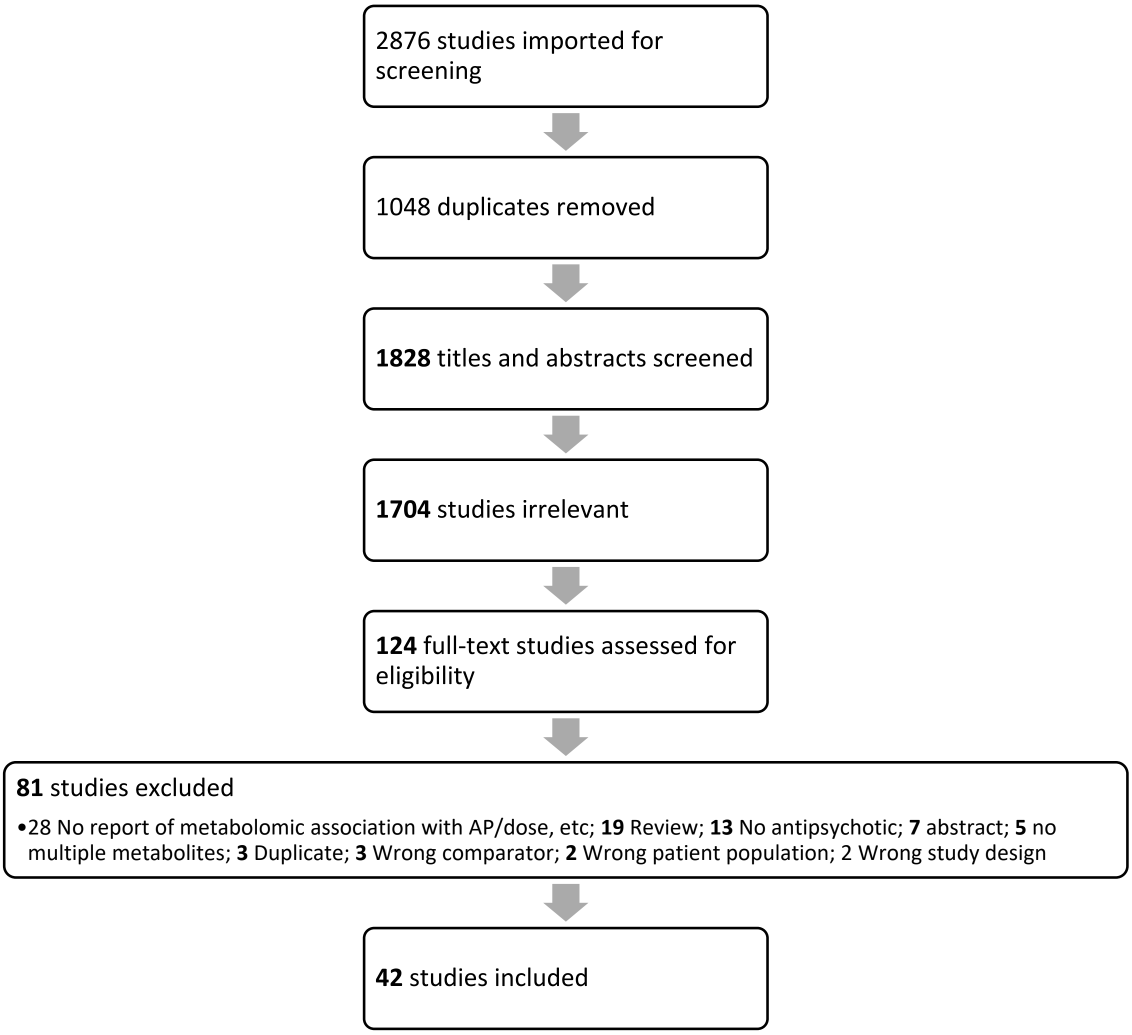

Table S1. Included Study Antipsychotic Characteristics, Age and Sex

Study	Antipsychotic Type	Sample Mean Age	% female
Al Awam 2015 [1]	85.2% atypical antipsychotic; 25.9% typical antipsychotic[a]	34.78	22.2
Aquino 2018[2]	31% olanzapine, 43% risperidone, 26% quetiapine	33.75	41
Atagün 2018[3]	51.2% on “antipsychotic”	36.85	55
Bicikova 2013[4]	45.4% olanzapine, 41% risperidone, 13.6% amisulpride	35 [b]	38
Brunkhorst-Kanaan 2019 [5]	51% antipsychotic	[c]	46
Buretić-Tomljanović 2008 [6]	32% quetiapine, 25% aripiprazole, 32% olanzapine, 11% risperidone, 9% sertindole, 7% ziprasidone, 7% perphenazine, 5% clozapine [a]	26.2	40
Cai 2012 [7]	risperidone	27.6	45
Cao 2019[8]	NR in detail (acknowledged a mix of typical and atypical antipsychotics)	28.91	57.4
Condray 2011[9]	70.1% risperidone, 20.8% olanzapine, 8.3% quetiapine(n=2), 4.2% aripiprazole, 4.2% haloperidol [a]	22.6	NR
deAlmeida 2020[10]	43% risperidone, 31% olanzapine and 26% quetiapine	37	41
Evans 2014[11]	clozapine, olanzapine, aripiprazole, risperidone, paliperidone, quetiapine, ziprasidone (proportions not reported)	45.0	73
Fujii 2017[12]	NR	45.0	58
He 2012[13]	12.1% haloperidol, 17.3% amisulpride, 17.8% clozapine, 17.3% olanzapine, and 17.8% risperidone	38.1	46.1
Kaddurah-Daouk 2007 [14]	40% olanzapine; 32% aripiprazole; 28% risperidone	30.9	18.5
Kim 2022[15]	NR	42.94	52.5
Kriisa 2017[16]	quetiapine 28.3%; aripiprazole 21.7%; olanzapine 26.1%; risperidone 4.3%; sertindole 4.3%; ziprasidone 6.5%; clozapine 4.3%; perphenazine 4.3%	NR	NR
Lenski 2021[17]	High proportion of antipsychotic polypharmacy (69.8% on >1 antipsychotic). Highest proportion of patients were on olanzapine and risperidone	44.8	52.1
Li 2022[18]	22.6% risperidone; 22.0% olanzapine; 8.6% aripiprazole; amisulpride 4.9%; clozapine 4.9%, others 7.0%	32.7	55
Liu 2015[19]	NR	28.5	55
Liu 2020[20]	Olanzapine	NR	NR
Liu 2021[21]	Olanzapine	27.4	100
Maes 2019[22]	NR in detail (only states "atypical")	42.7	19
McEvoy 2013[23]	NR in detail (randomized to risperidone or aripiprazole)	31.8	27.5
Mednova 2021[24]	NR	35	48.7
Mednova 2022[25]	NR	35.5	49.6
Okamoto 2021 [26]	13.3% risperidone, 13.3% olanzapine, 11.1% levomepromazine, 11.1% clozapine, 11.1% aripiprazole, 8.9% haloperidol, 6.7% quetiapine, 6.7% zotepine, 6.7% brexpiprazole, 4.4% blonanserin, 4.4% asenapine, 2.2% fluphenazine[a]	48.0	43
Orešič 2011[27]	8.6% AAP; 38.8% typical AP	54.4	53.5
Paredes 2014[28]	36.7% risperidone, 33.3% olanzapine, 16.7% ziprasidone, 8.3% quetiapine, 3.3% clozapine, 1.7% aripiprazole	42.7	25
Parksepp 2020[29]	NR	27	40
Parksepp 2022[30]	NR	26.6	43
Qiao 2016[31]	olanzapine	28.3	100
Schwarz 2008[32]	NR brain; Blood clozapine	41.7	26.7
Suvitaival 2016[33]	33% olanzapine, 28% risperidone	24.5	44

Tessier 2016 [34]	NR in detail; mix of typical (haloperidol, chlorpromazine, flupentixol) and atypical (amisulpride, aripiprazole, clozapine, olanzapine, quetiapine, sertindole and risperidone).	43.8	35.2
Tkachev 2021[35]	NR in detail (mix of typical and atypical)	range 17-43	58
Wang 2018[36]	8.3% haloperidol; 0.92% chlorpromazine; 3.7% promethazine; 18.4% olanzapine; 31.2% risperidone; 27.5% clozapine; 38.5% quetiapine; 22% ziprasidone; 15.6% aripiprazole; 3.7% perphenazine, 1.8% Sulpride	29	55.7
Wang 2022[37]	olanzapine	27.4	100
Wood 2015 [38]	NR in detail (atypical antipsychotics)	47	22
Xuan 2011[39]	risperidone	41	44
Yan 2018[40]	60% haloperidol, 45% quetiapine, 40% risperidone, 25% olanzapine, 15% clozapine[a]	32.2	45
Yao 2010a[41]	65% risperidone, 15.4% olanzapine, 7.7% quetiapine, 7.7% haloperidol, 3.8% aripiprazole[a]	23.9	24
Yao 2010b[42]	65% risperidone, 15.4% olanzapine, 7.7% quetiapine, 7.7% haloperidol, 3.8% aripiprazole[a]	23.9	24

[a]cumulative >100% due to polypharmacy

[b]median

[c]only distributions reported, please see paper

NR: not reported

Table S2. Detailed Findings of Untargeted Metabolomic Studies

Study	Summary of significant metabolite, or metabolite class, associations with antipsychotic treatment
Al Awam 2015 [1]	1-Oxo-proline, 2-piperidinec carboxylic acid, 6-deoxy-mannofuranose, galactose oxime, oleic acid, pentadecanoic acid, heptadecanoic acid, eicosanoic acid, cholesterol
Cai 2012 [7]	LPC(16:0), LPC (18:2), LPC (18:1), LPC (18:0), PC (16:0/18:2), uric acid, pregnanediol, lipoprotein, 3-HB, lactate, acetoacetate, glucose, glycine, LDL, VLDL, HDL, UFA, creatine, valine, glycine, glucose, taurine, and TMAO.
Cao 2019[8]	Oleoylcarnitine, l-palmitoylcarnitine, linoleyl carnitine, l-acetylcarnitine, LysoPCl(16:0), LysoPC(15:0), LysoPC(14:0), 2,5-Dichloro-4-oxohex-2-enedioate, L-proline, D-glutamic acid, L-arginine, L-lysine, Ornithine, and L-cysteine
Fujii 2017[12]	3-Methylhistidine and morpholine[a]
Kim 2022[15]	None identified
Liu 2020[20]	14 amino acids, 9 LysoPEs, 3 lipid/fatty acids, 2 neurotransmitters, 2 nucleotide-type species, 1 PCs
Liu 2021[21]	175 differential metabolites with most upregulated metabolites included LysoPE (20:3), LysoPE (16:1), LysoPC(14:0) and LysoPC(22:2) and most downregulated metabolites included carnitine and other organic esters.
Okamoto 2021 [26]	γ-Glu-Trp, γ-Glu-His, γ-Glu-Val, γ-Glu-Phe, γ-Glu-Ile, γ-Glu-Leu, Urea, N-acetylalanine, Isethionic acid, Creatinine, Hydroxyindole, Tetrahydrouridine, Isatin, N2- Phenylacetylglutamine, Piperidine, glutamyl-glutamate, Melamine, N2-acetylaminoadipic acid, Lipoamide
Orešič 2011[27]	Antipsychotic use was associated with an increase in an 18 specie metabolomic cluster containing ketone bodies and free fatty acids and a decrease in a 53 specie metabolomic cluster containing energy metabolites and various organic acids. The study performed analyses on clusters and not individual metabolites.
Paredes 2014[28]	Antipsychotics with low risk of metabolic side effects showed significant differences compared to controls for malate and alpha-ketoglutarate. Antipsychotics with high risk for metabolic side effects showed significance differences compared to controls for 2-hydroxyglutarate, glutamate and kyneurine
Qiao 2016[31]	13 metabolites that were identified as different between case and control were investigated for changes before and after antipsychotic treatment. Eight of the 13 metabolites had significant changes with treatment including for phytosphingosine, l-methionine, LPC (20:3), N-acetylglutamic acid, LPC (14:0), 2-oxovaleric acid, O-acetylserine and PE (P−16:0/0:0)
Suvitaival 2016[33]	An effect of olanzapine treatment was observed on a carboxylic acid metabolite cluster containing 11 metabolites.
Wang 2022[37]	After antipsychotic treatment 2-Octenoylcarnitine decreased while significant increases were observed for linoelaidyl carnitine, 9-Decenoylcarnitine and 11Z-Octadecenylcarnitine. Additionally, linoelaidyl carnitine was found to be different at baseline between responders and non-responders and also predicted response to olanzapine.
Xuan 2011[39]	20 metabolites were significantly associated with antipsychotic treatment and included amino acids, fatty acids and other compounds. Of these 20, 14 were associated with being an antipsychotic responder while 8 were associated with being an antipsychotic non-responder. 20 metabolites: Glucose, 1,3-bisphosphoglycerate, lactate, uric acid, γ-tocopherol, aspartate, glycine, tryptophan, phenylalanine, tyrosine, myo-inositol, glucuronic acid, linoleic acid, oleic acid, stearic acid, palmitic acid, glycerol, cholesterol, lactobionic acid, erythrose

TMAO: Trimethylamine N-oxide

[a]Significant correlation with lifetime quantity of fluphenazine or equivalent

Table S3. Detailed Findings of Untargeted Lipidomic Studies

Study	Number of lipidomic features identified/ion signals	Findings with antipsychotic treatment
Al Awam 2015 [1]	62	The following lipid metabolites had a >70% diagnostic value for differentiating patients on antipsychotics with healthy controls; oleic acid, pentadecanoic acid, heptadecanoic acid, eicosanoic acid, cholesterol, Ethoxy- cholest-5-ene, Ethoxy- cholest-5-ene, cholest-5-en-3-ol, Cholesta-3,5-diene
Aquino 2018[2]	1600	Lipidomic profiles were compared between responders and non-responders and 147, 148 and 193 significant lipids were found for olanzapine, risperidone and quetiapine, respectively. Amongst these compounds, PE(18:0/20:3), 2-amino-heptanoic acid (S and R forms) and PC(16:0/20:4) were common between the antipsychotics. Significant compounds with olanzapine treatment included 64 PCs, 31 PSs, 30 Pes, 7 SMs, 4 PAs, 1 cer and various other lipid compounds. For risperidone this included 52 PCs, 48 Pes, 24 PGs, 16 Pis, 3 PAs, and various other lipid compounds. Finally, for quetiapine significant compounds included 64 PCs, 58 Pes, 16 PSs, 14 Pis, 10 PGs, 4 PAs, 3 SMs and various other lipids.
deAlmeida 2020[10]	1590	PCA showed separation between baseline and post-treatment group which could be further separated based on good versus poor response. Risperidone response was associated with effects on the largest number of lipid classes followed by olanzapine. Quetiapine response was associated with effects on the fewest lipid classes. For risperidone, decreases in poor responders were observed for PGs, PCs, PSs, SMs, Cers, and DGs and increases in poor responders were observed for TGs. Risperidone good responders had decreases in PCs and PIs and increases in PCs and Pes. For olanzapine, good responders had increases in PGs, PCs and PAs and poor responders had increases in PGs and decreases in PSs and PAs.
Paredes 2014[28]	205	DGs and TGs were increased compared to controls but did not reach statistical significance. Medium metabolic risk antipsychotics showed significant differences in DG 35:2, DG 42:2, and TG 52:1 compared to controls. DG 42:2 was also significantly increased in the high metabolic risk antipsychotic group.
Schwarz 2008[32]	NR	In brain samples: grey matter PCs were higher in antipsychotic-treated versus non-treated and healthy controls. No effect on white matter PCs or Cers and FFAs in either grwy or white matter. In RBC: Stearic acid decreased and Cer 34:1 increased after antipsychotic treatment
Suvitaival 2016[33]	1148	Baseline length of antipsychotic treatment significantly associated with two lipid clusters consisting of a total of 90 TGs. After olanzapine treatment lipdomic levels mostly increased and particularly in two lipid clusters (one cluster of 45 PCs and Pes and one cluster of 45 PCs). After risperidone treatment lipidomic profiles decreased overall with the biggest decrease in in three bi- and monounsaturated TG clusters.
Tkachev 2021[35]	322	TAG 42:0, TAG 44:0, and TAG 44:1 were significantly associated with worst responders to several medication including antipsychotics. These changes were significantly greater in worst responders versus best responders.
Yan 2018[40]	445	A total of 50 lipids were significantly decreased following antipsychotic treatment which included Ces, TGs, Fas, GlcCers, LysoPCs, PCs, plasmenyl-PCs, SMs and Cers. The lipid classes containing p-PE, LysoPE and acylcarnitines were not influenced by 5antipsychotic treatment.

Abbreviations: Cer=ceramide; FA=fatty acyl; FFA=free fatty acids; GL=glycerolipid; GPL=glycerophospholipid; PA=phosphatidic acids; PC=phosphatidylcholine; PE=phosphatidylethanolamine; PG = Phosphatidylglycerol; PI=phosphatidylinositol; PS=phosphatidylserine; SM=sphingomyelin; SL=sterol lipid; TAG=triacylglycerol

Table S4. Detailed Findings of Targeted Metabolomic Studies

Study	# metabolites	Metabolites Targeted	Results
Atagün 2018[3]	6	Neurotransmitters /Sugars including glutamate, glutamate+glutamine, inositol containing compounds, creatine +phosphocreatine, choline containing compounds, n-Acetyl Aspartate	No correlations observed with CPZEs
Bicikova 2013[4]	31	Steroids including 16-Hydroxypregnenolone, 17-Hydroxypregnenolone, 20-Dihydropregnenolone, 20-Dihydroprogesterone, 5,20-Tetrahydroprogesterone, 5-Androstane-3,17-Diol, 5-Dihydroprogesterone, 5-Dihydrotestosterone, 5-Pregnane-3,20-diol, Allopregnanolone, Androstenediol, Androstenedione, Androsterone, Cortisol, DHEA, DHEAS, Epiandrosterone, Epietiocholanolone, Epipregnanolone, Etiocholanolone, Isopregnanolone, Pregnanolone, Pregnenolone, Pregnenolone sulfate, Progesterone, Testosterone	In males, an increase in androsterone was observed following treatment. In females, a decrease in 5,20-yetrahydroprogesterone, etiocholanolone and pregnenolone sulfate was observed following treatment.
Cai 2012 [7]	11	Neurotransmitters including Glutamate, gaba-aminobutyrate, glutamine, Dopamine, norepinephrine, 5-hydroxytryptamine, 3,4-dihydroxyphenylacetic acid, homovanillic acid, vanilmandelic acid, 3-methoxy-4-hydroxyphenylglycol and 5-hydroxytindole3-acetic acid.	Multivariate analysis of plasma and urine neurotransmitterss by PLSD-DA showed good separation between baseline and 6-week treated patient. Shifts included ↑ glutamate, ↑ glutamine, ↓ dopamaine, ↑ dihydroxyphenylacetic acid, ↑ homovanillic acid, ↑ norepinephrine, ↑ vanillylmandelic acid, ↓ 3-methoxy-4-hydroxyphenylglycol, ↑ 5-hydroxytryptamine, ↓ 5-hydroxyindoleacetic acid
Condray 2011[9]	7	Tryptophan metabolites including Tryptophan; 5-HT, serotonin; 5-HIAA, 5-hydroxyindoleacetic acid; melatonin; kynurenine ; 3-hydroxykynurenine; tryptamine.	No metabolite significantly changed before and after treatment. 3-hydroxykynurenine were associated with symptom improvement on antipsychotics.
He 2012[13]	163	AbsoluteIDQ® p150 kit covers 4 compound classes (acyl carnitines, amino acids, glycerophospho- and sphingolipids and hexose)	Only PC acyl-akyl C34:3 was significantly different between Antipsychotic free and antipsychotic-treated patients. However, several 5 metabolites showed difference between baseline and healthy controls (increased ornithine and decreased arginine, glutamine, histidine and PC acyl-akyl C38:6).
Kriisa 2017[16]	206	AbsoluteIDQ® p180 kit covers 21 amino acid, 21 biogenic amines, hexose, 40 acylcarnitines, 14 lysophosphatidylcholines, 76 phosphatidylcholines, 15 sphingolipids but investigators only included acylcarnitines in results. Also used the Randox biochip which includes 12 cytokine and growth factors (TNF-α, IFN-γ, IL1α, IL-1β, IL-2, IL-4, IL-6, IL-8, IL-10, MCP-1, VEGF, EGF, C-peptide, insulin leptin, resistin, ferritin, and PAI-1.	Metabolites associated with the main effect of treatment from linear models not corrected for multiple testing included 13 ACs, IL-2, IL-4, INF-γ, C16_hexadecanoyl-carnitine , C18:1_ octadecenoyl-carnitine , C18:2_octadecadienyl-carnitine, C3_propionyl-carnitine).<br>EGF, C-peptide, and leptin.<br><br>4 pre-post metabolites survived multiple testing correction: ↓ C16_hexadecanoyl-carnitine , ↓ C18:1_ octadecenoyl-carnitine , ↓ C18:2_Octadecadienyl-carnitine, ↑ C3_Propionyl-carnitine<br><br>13 ACs included C14_Tetradecanoylcarnitine, C14:1_Tetradecanoylcarnitine, C14:1-OH_Hydroxytetradecenoylcarnitine, C14:2_Tetradecadienylcarnitine, C16_Hexadecanoylcarnitine, C16:1_Hexadecenoylcarnitine, C16:1-OH_Hydroxyhexadecenoylcarnitine, C18_Octadecanoylcarnitine, C18:1_Octadecenoylcarnitine, C18:1-OH_Hydroxyoctadecenoylcarnitine, C18:2_Octadecadienylcarnitine, C3_Propionylcarnitine, C3-DC(C4-OH)_ Malonylcarnitine(Hydroxybutyrylcarnitine)
Lenski 2021[17]	220	Various polar metabolite classes including amino acids, organic acids, amines and amides and sugars	Significant metabolites pre to post included 5 amino acids, 6 acylcarnitines, 4 carboxylic acids, 1 catecholamine, 1 nucleoside, 1 pyridine, and 1 tetrapyrrole.<br><br>Individual significant metabolites included: ↑3-hydroxy-3-methylglutarate, ↓6-hydroxydopamine, ↑Biliverdin, ↓Creatine, ↑Decanoylcarnitine, ↑Deoxyuridine, ↑D-galacturonic acid, ↑Hexanoylcarnitine, ↑Hydroxypyruvate, ↓Kynurenine, ↓Kynurenine/Tryptophan ratio, ↓l-alanine, ↑Lauroylcarnitine, ↑Malonate, ↓N-Amidino-l-Aspartate,

			↑Octanoylcarnitine, ↑Oleylcarnitine, ↓Propionylcarnitine, ↓Pyridoxamine, ↑Thyroxine
Liu 2015[19]	13	Glucose metabolism pathway metabolites. Metabolites included: glucose, glucose 6-phosphate, fructose 6-phosphate, fructose, glyceraldehyde-3-phosphate, dihydroxyacetone phosphate, glycerol 3-phosphate, glycerate 3-phosphate, pyruvate, lactic acid, citric acid, succinic acid, ribose 5-phosphate	Ribose 5-phosphate was significantly increased between treated and untreated patients.
Liu 2020[20]	13	NTs including L-tryptophan, L-tyrosine, glutamine, L-asparagine, acetylcholine, 5-hydroxytryptophan, 5-hydroxyindoleacetic acid, 5-hydroxytryptamine, g-aminobutyric acid, glutamate, L-3,4-dihydroxyphenylalanine, taurine, kynurenine	Significant decreases in L-tryptophan, L-tyrosine, 5-hydroxytryptophan, 5-hydroxyindoleacetic acid, g-aminobutyric acid, L-3,4-dihydroxyphenylalanine, taurine, kynurenine after treatment with olanzapine
Maes 2019[22]	6	Nitro-oxidative and nitrosative stress pathways. 6 products including lipid hydroperoxides, nitric oxide, malondialdehyde, catalase, superoxide dismutase and advanced oxidation protein	Antipsychotics had no effect on the z-scores between the patient groups.
Mednova 2021[24]	45	Amino Acids and acylcarnitines including alanine, arginine, aspartate, citrulline, glycine, methionine, ornithine, phenylalanine, tyrosine, valine, leucine/isoleucine, proline, alanine, arginine, aspartate, C0, C2, C3, C3-DC, C4, C4-OH, C4-DC, C5, C5-OH, C5:1, C5-DC, C6, C8, C8:1, C10, C10:1, C12, C14, C14-OH, C14:1, C14:2, C16, C16-OH, C16:1, C16:1-OH, C18, C18-OH, C18:1, C18:1-OH, C18:2-OH	8 amino acids and 12 acylcarnitines were significantly different between patients treated with antipsychotics and healthy controls. These significant metabolites included: ↓ L-tryptophan, ↓ L-tyrosine, ↓ 5-hydroxytryptophan, ↓5-hydroxyindoleacetic acid, ↓ g-aminobutyric acid, ↓ 4 dihydroxyphenylalanine, ↓ taurine, ↓ kynurenine, ↓ Arginine, ↓ Aspartate, ↓ Citrulline, ↓ Glycine, ↓ Ornithine, ↓ Valine, ↑ Arginine, ↓ Aspartate, ↑ C4-DC, ↓ C5:1, ↓ C14, ↓ C14-OH, ↓ C16-OH, ↓ C16:1, ↓ C16:1-OH, ↓ C18, ↓ C18-OH, ↓ C18:1, ↓ C18:1-OH, ↓ C18:2-OH
Mednova 2022[25]	32	Amino Acids and acylcarnitines including C0, C2, C3, C3-DC, C4, C4-OH, C4-DC, C5, C5-OH, C5:1, C5-DC, C6, C8, C8:1, C10, C10:1, C12, C14-OH, C14:1, C14:2, C16, C16-OH, C16:1, C16:1-OH, C18, C18-OH, C18:1, C18:1-OH, C18:2-OH, alanine, valine, leucine/isoleucine	Five metabolites were significantly different between healthy controls and those on antipsychotics with metabolic syndrome (↓ C10, ↓ C10:1, ↓ C12, ↓ C18, ↑ Alanine). Six metabolites were significantly different between healthy controls and those on antipsychotics without metabolic syndrome (↓ C5, ↓ C5:1, ↓ C10, ↓ C10:1, ↓ C12, ↓ C18).
Parksepp 2020[29]	31	AbsoluteIDQ® p180 kit to measure only amino acids (21) and biogenic amines (10) including alanine, arginine, asparagine, aspartate, citrulline, glutamine, glutamate, glycine, histidine, isoleucine, leucine, lysine, methionine, ornithine, phenylalanine, proline, serine, threonine, tryptophan, tyrosine, valine, acetylornithine, alpha-aminoadipic acid, asymmetric dimethylarginine, creatinine, histamine, kynurenine, putrescine, serotonin, symmetric dimethylarginine, taurine. The lipid levels obtained by the kit were not utilized in the analysis.	Six months of antipsychotic treatment returned all metabolites to control levels. Five years of treatment caused significant changes in 9 metabolites and 4 of 5 metabolite rations calculated. Metabolite changes at 6 months included: ↑ Methionine, ↓ Alpha- amino-adipic acid, ↓ Alpha-amino-adipic acid / Kynurenine. Metabolite changes at 5 years included: ↓ Aspartate, ↑ Glutamine, ↓ Glutamate, ↑ Valine, ↓ Alpha-amino-adipic acid, ↓ Histamine, ↑ Putrescine, ↑ Taurine, ↓ Alpha-amino-adipic acid / Kynurenine, ↓ Aspartate / Asparagine, ↓ Glutamate / Glutamine, ↑ Ornithine / Arginine.
Yao 2010a[41]	6	Purine pathway metabolites including uric acid, xanthine, xanthosine, guanine, guanosine, and hypoxanthine. Ratios also analyzed.	Guanine and the uric acid to guanosine ratio were the only significant metabolites after treatment compared to baseline.
Yao 2010b[42]	13	Tryptophan pathway metabolites including 5-hydroxyindoleacetic acid, serotonin, 5-hydroxytryptophan, kynurenine, melatonin, N-acetyl serotonin, N-methylserotonin, 3-hydroxyanthranillic acid, 3-hydroxykynurenine; tryptophol, tryptophan, tryptamine. Ratios also analyzed.	No metabolites or ratios were significantly different after treatment compared to baseline. Only melatonin was significant after treatment compared to healthy controls.

CPZEs:, DHEA:, IL:, IN:, TNF:, MCP: VEGF, EGF,

Table S5. Detailed Findings of Targeted Lipidomic Studies

Study	# lipid metabolites	Lipid metabolites Targeted	Results
Brunkhorst-Kanaan 2019 [5]	36	Sphingolipids and ceramides, lysophosphatidic acids, endocannabinoids including sphingosine sphinganine, sphingosine-1-phosphate sphinganine-1-phosphate, c16 dihydroceramide, c18 dihydroceramide C24 Dihydroceramide, C24:1, dihydroceramide, C14 Cer, C16 Cer, C18 Cer, C20 Cer, C24 Cer, C24:1 Cer, C16 GluCer, C18 GluCer, C18:1 GluCer, C24:1 GluCer, C16 LacCer, C18 LacCer, C24 LacCer, C24:1 LacCer, LPA 16:0, LPA 18:0, LPA 18:1, LPA 18:2, LPA 18:3, LPA 20:0, LPA 20:4, arachidonoyl ethanolamide,palmitoyl ethanolamide, oleoyl ethanolamide, 1-arachidonoyl glycerol, 2-arachidonoyl glycerol, 6-biopterin, d-neopterin	No effect of antipsychotics as a class on lipid metabolites. Olanzapine was associated with significantly increased ceramides as a class. The individually increased ceramides with olanzapine included ↑C16Cer, ↑C20Cer, ↑C22Cer, ↑C24Cer and ↑C24:1Cer.
Buretić-Tomljanović 2008 [6]	105	Absolute IDQ® p150 kit including 90 Glycerophospholipids and 15 sphingomyelins* (investigators did not include other metabolites on kit in analysis)	Antipsychotic treatment significantly increased two Lysophosphatidylcholine acyls, Total phosphatidylcholine diacyls, and 9 phosphatidylcholines while treatment significantly decreased two sphingomyelins.<br><br>These lipid changes included; ↑LysoPCa-C14:0, ↑LysoPC-a-C20:3, ↑PCaa-C32:2, ↑PC-aa-C34:3, ↑PC-aa-C34:4, ↑PC-aa-C36:1, ↑PC-aa-C36:2, ↑PC-aa-C36:3, ↑PC-aa-C36:6, ↑PC-aa-C38:3, ↑PC-aa-C40:5, ↓SM-(OH)-C16:1, ↓SM-C18:0
Evans 2014[11]	16	Eight n-3 and n-6 polyunsaturated fatty acids including alpha-linolenic acid, eicosapentaenoic acid, docosapentaenoic acid, docosahexaenoic acid, linoleic acid, eicosadienoic acid,,gamma linolenic acid, dihomogamma linolenic acid, arachidonic acid, 13(S)-Hydroperoxylinoleic acid, 9,10-epoxy-octadecenoic acid, 15(S)-Hydroxyeicosatetraenoic Acid, leukotriene A4, Prostaglandin E1, Prostaglandin F2alpha, Prostaglandin G2	Atypical antipsychotic use positively correlated with eicosapentaenoic acid and gamma linolenic acid.
Kaddurah-Daouk 2007 [14]	>300	Seven lipid classes including ceramides, diacylglycerols, fatty acids, lyso-lipids, phosphatidylcholines, phosphatidylethanolamines, and tryiglycerides	At the level of lipid class, increases in phosphatidylcholines, phosphatidylethanolamines, diacylglycerols and tryiglycerides and a decrease in fatty acids were observed with antipsychotic treatment. Thirty-four individual lipids changed with antipsychotic treatment. Specific changes included: ↓ CE14.1n5, ↓ CE18.3n6, ↓ CE18.3n3, ↓ DG22.1n9, ↑ FA22.4n6, ↓ LY18.0, ↓ LY20.4n3, ↓ PC14.0, ↓ PC18.0, ↓ PC18.3n6, ↓ PC20.3n6, ↓ PC18.3n3, ↓ PC20.4n3, ↓ PC20.5n3, ↓ PCdm16.0, ↑ PE22.0, ↓ PE18.1n9, ↓ PE20.3n9, ↓ PE18.2n6, ↓ PE20.3n6, ↓PE20.4n6, ↓ PE22.4n6, ↓ PE22.5n3, ↓ PEdm16.0, ↓ PEdm18.0, ↓ PEdm18.1n7, ↓ PEdm18.1n9, ↓ PELC, ↓ PEMUFA, ↓ PEPUFA, ↓ PEn6, ↓ PEn9, ↓ PEdm, ↓ TG20.4n3.<br>Only phosphatidylethanolamine-polyunsatu-rated fatty acids (PE-PUFAs), PE-n6 fatty acid family and PE levels increased with all 3 antipsychotics studies. The remaining lipid metabolite effect varied by antipsychotic.
Li 2022[18]	10	Fatty acids including C16:0, C18:0, C18:1n9c, C18:2n6c, C20:3n6, C20:4n6, C20:5n3, C22:4n6, C22:5n3 and C22:6n3	All fatty acids increased after antipsychotic treatment. Individual fatty acid increases included: C16:0, C18:0, C18:1n9c, C18:2n6c, C20:3n6c, C20:4n6c, C22:4n6c, C20:5n3, C22:5n3, C22:6n3. Additionally, class increases were observed for total fatty acids, saturated fatty acids, and polyunsaturated fatty acids after treatment.
McEvoy 2013[23]	4	PC.n3, PC.n6, PE.n3, PE.n6	PE.n3 and PE.n6 increased following treatment in first episode patients. No changes were observed after treatment in recurrent episode patients. No class-wide changes observed for pre to post

			treatment. Ratios of lipid products (inidication of enzymatic steps in lipid biosynthesis) showed significant decreases for recurrent episode patients, pre to post treatment for: PC 22:6n3/22:5n3, PC 20:4n6/20:3n6, and PE 20:4n6/20:3n6.
Parksepp 2022[30]	55	eCBs and eCB-like compound and glycerophospholipids	Significant changes after 5 years of treatment included: 1 eCB was increased, 2 eCB ratios decreased, 4 PCs increased and 9 PCs decreased. No significant changes observed at 6 months.<br><br>Changed eCBs included ↑Arachidonoylglycerol (2-AG) and ratios included linoleoylethanolamide/arachidonoylglycerol and linoleoylethanolamide/ anandamide.<br><br>Increased PCs included PC aa C38:1, PC aa C38:5, PC aa C38:6, PC aa C40:6. Decreased PCs included PC aa C30:2, PC aa C36:0, PC aa C40:1, PC aa C40:3, PC aa C40:4, PC aa C42:0, PC aa C42:1, PC aa C42:2, PC aa C42:5, PC aa C42:6.
Tessier 2016 [34]	128	45 phosphatidylcholines, 18 phosphatidylserines, 23 sphingomyelins, 42 phosphatidylethanolamines	Percent composition of sphingomyelins were significantly decreased and phosphatidylserines were increased in antipsychotic treated patients compared to healthy controls. Individual specie analyses comparing concentrations not provided.
Wang 2018[36]	49	eicosanoids and related compounds	A total of 22 metabolites were significantly altered after treatment compared to baseline which included 9 arachidonic acid eicosanoids, 7 linoleic acid eicosanoids, 1 eicosapentaenoic acid eicosanoid, 2 docosahexaenoic acid related eicosanoids, 2 ethanolamide metabolites, and 1 eicosadienoic acid related eicosanoid.<br><br>These significantly changed metabolites included ↑PGE2, ↑PGF2α, ↑TXB2, ↑11-dehydro-TXB2, ↓11,12-DHET, ↓14,15-DHET, ↓20-carboxy-AA, ↓5-KETE, ↑ 12-HETE, ↓4-HDoHE, ↓7-HDoHE, ↑12-HEPE, ↓AEA, ↓ OEA, ↓15-KEDE, ↓9-HpODE, ↓9-HODE, ↓9-KODE, ↓13-HpODE, ↓13-HODE, ↓13-KODE, ↓9,10-DiHOME
Wood 2015 [38]	15	7 choline plasmalogens, 3 lysoplasmalogens, 4 ethanolamine plasmalogens and DHA.<br>Choline plasmalogens included 34:1, 34:2, 34:3, 36:1, 36:2, 36:5, 40:6.<br>Lysoplasmalogens included LPC 18:0, LPE 16:0, LPE 18:1.<br>Ethanolamine plasmalogens included 34:2, 36:5, 38:6 and 40:6.	In the plasma all choline and ethanolamine plasmalogens and DHA were decreased in antipsychotic treated patients versus healthy controls. In platelets, choline plasmalogens were increased while DHA and ethanolamine plasmalogens were decreased compared to healthy controls.<br>The specific choline plasmalogens changes in plasma included ↓PC34:1, ↓PC34:2(↑ platelets), ↓PC34:3 (↑ platelets), ↓PC36:1, ↓PC36:2, ↓PC36:5 and ↑PC40:6 (platelets only). The specific ethanolamine plasmalogens included ↓PE34:2, ↓PE36:5 (platelets only), ↓PE38:6 and ↓PE40:6.

Abbreviations: AA= arachidonic acid; Cer=ceramide; DHA= docosahexaenoic acid; EA= ethanolamide; ECB=endocannabinoid; EDA= eicosadienoic acid; EPA= eicosapentaenoic acid; FA=fatty acyl; GL=glycerolipid; GPL=glycerophospholipid; LA= linoleic acid; PA=phosphatidic acid; PC=phosphatidylcholine; PE=phosphatidylethanolamine; PG = Phosphatidylglycerol; PI=phosphatidylinositol; PS=phosphatidylserine; SM=sphingomyelin; SL=sterol lipid

Quality Assessment Supplementary Tables 6 – 10

Table S6. Quality Assessment of Cross-Sectional Studies using NHLBI Quality Assessment Tool.

	Liu 2015[19]	Maes 2019[22]	Mednova 2021[24]	Mednova 2022[25]	Orešič 2011[27]	Paredes 2014[28]	Schwarz 2008[32]	Tessier 2016 [34]	Wood 2015 [38]
1. Was the research question or objective in this paper clearly stated?	Yes	Yes	Yes	Yes	Yes	Yes	Yes	Yes	Yes
2. Was the study population clearly specified and defined?	Yes	Yes	Yes	Yes	Yes	Yes	Yes	Yes	Yes
3. Was the participation rate of eligible persons at least 50%?	CD	Yes	CD	Yes	Yes	Yes	Yes	CD	CD
4. Were all the subjects selected or recruited from the same or similar populations (including the same time period)? Were inclusion and exclusion criteria for being in the study prespecified and applied uniformly to all participants?	Yes	Yes	Yes	Yes	Yes	Yes	Yes	Yes	Yes
5. Was a sample size justification, power description, or variance and effect estimates provided?	No	No	No	No	Yes	Yes	Yes	Yes	No
6. For the analyses in this paper, were the exposure(s) of interest measured prior to the outcome(s) being measured?	No	No	No	No	No	No	No	No	No
7. Was the timeframe sufficient so that one could reasonably expect to see an association between exposure and outcome if it existed?	Yes	Yes	Yes	Yes	Yes	Yes	Yes	Yes	Yes
8. For exposures that can vary in amount or level, did the study examine different levels of the exposure as related to the outcome (e.g., categories of exposure, or exposure measured as continuous variable)?	NA	Yes	Yes	Yes	Yes	Yes	NA	Yes	Yes
9. Were the exposure measures (independent variables) clearly defined, valid, reliable, and implemented consistently across all study participants?	Yes	Yes	Yes	Yes	Yes	Yes	Yes	Yes	Yes
10. Was the exposure(s) assessed more than once over time?	No	No	No	No	No	No	No	No	No
11. Were the outcome measures (dependent variables) clearly defined, valid, reliable, and implemented consistently across all study participants?	Yes	Yes	Yes	Yes	Yes	Yes	Yes	Yes	Yes
12. Were the outcome assessors blinded to the exposure status of participants?	NR	NR	NR	NR	NR	NR	NR	NR	NR
13. Was loss to follow-up after baseline 20% or less?	NA	NA	NA	NA	NA	NA	NA	NA	NA
14. Were key potential confounding variables measured and adjusted statistically for their impact on the relationship between exposure(s) and outcome(s)?	No	Yes	No	No	Yes	Yes	No	Yes	No

Possible answers include: Yes, No, Not Applicable (NA), Not Reported (NR), Cannot Determine (CD) or Partial (P). For instructions and details of quality assessment tool see: https://www.nhlbi.nih.gov/health-topics/study-quality-assessment-tools

Table S7. Quality Assessment of Case-Control Studies using NHLBI Quality Assessment Tool

	Al Awam 2015 [1]	Atagün 2018[3]	Brunkhorst-Kanaan 2019 [5]	Evans 2014[11]	Fujii 2017[12]	He 2012[13]	Okamoto 2021 [26]
1. Was the research question or objective in this paper clearly stated and appropriate?	Yes	Yes	Yes	Yes	Yes	Yes	Yes
2. Was the study population clearly specified and defined?	Yes	Yes	Yes	Yes	Yes	Yes	Yes
3. Did the authors include a sample size justification?	No	No	No	No	No	No	NR
4. Were controls selected or recruited from the same or similar population that gave rise to the cases (including the same timeframe)?	Yes	NR	Yes	Yes	CD	Yes	Yes
5. Were the definitions, inclusion and exclusion criteria, algorithms or processes used to identify or select cases and controls valid, reliable, and implemented consistently across all study participants?	NR	Yes	Yes	Yes	NR	Yes	Yes
6. Were the cases clearly defined and differentiated from controls?	Yes	Yes	Yes	Yes	Yes	Yes	Yes
7. If less than 100 percent of eligible cases and/or controls were selected for the study, were the cases and/or controls randomly selected from those eligible?	NR	NR	NR	NR	NR	NR	NR
8. Was there use of concurrent controls?	Yes	CD	Yes	Yes	CD	Yes	Yes
9. Were the investigators able to confirm that the exposure/risk occurred prior to the development of the condition or event that defined a participant as a case?	Yes	Yes	Yes	Yes	Yes	Yes	yes
10. Were the measures of exposure/risk clearly defined, valid, reliable, and implemented consistently (including the same time period) across all study participants?	Yes	Yes	Yes	Yes	Yes	Yes	yes
11. Were the assessors of exposure/risk blinded to the case or control status of participants?	NR	NR	NR	NR	NR	NR	NR
12. Were key potential confounding variables measured and adjusted statistically in the analyses? If matching was used, did the investigators account for matching during study analysis?	NR	NR	Yes	Yes	Yes	Yes	Yes

Possible answers include: Yes, No, Not Applicable (NA), Not Reported (NR), Cannot Determine (CD) or Partial (P). For instructions and details of quality assessment tool see: https://www.nhlbi.nih.gov/health-topics/study-quality-assessment-tools

Table S8. Quality Assessment of Pre-Post Studies using NHLBI Quality Assessment Tool.

	Aquino 2018[2]	Bicikova 2013[4]	Buretić-Tomljanović 2008 [6]	Cai 2012 [7]	Cao 2019[8]	Condray 2011[9]	deAlmeida 2020[10]	Kaddurah-Daouk 2007 [14]	Kim 2022[15]	Kriisa 2017[16]	Lenski 2021[17]	Li 2022[18]	Liu 2020[20]
1. Was the study question or objective clearly stated?	Yes	Yes	Yes	Yes	Yes	Yes	Yes	Yes	Yes	Yes	Yes	Yes	Yes
2. Were eligibility/selection criteria for the study population prespecified and clearly described?	Yes	Yes	Yes	Yes	Yes	Yes	Yes	Yes	Yes	Yes	Yes	Yes	No
3. Were the participants in the study representative of those who would be eligible for the test/service/intervention in the general or clinical population of interest?	Yes	Yes	Yes	Yes	Yes	Yes	Yes	Yes	Yes	Yes	Yes	Yes	Yes
4. Were all eligible participants that met the prespecified entry criteria enrolled?	Yes	Yes	Yes	No	Yes	No	Yes	Yes	CD	No	Yes	Yes	Yes
5. Was the sample size sufficiently large to provide confidence in the findings?	No	No	No	No	Yes	No	No	No	No	No	Yes	Yes	No
6. Was the test/service/intervention clearly described and delivered consistently across the study population?	Yes	Yes	Yes	Yes	Yes	Yes	Yes	Yes	Yes	Yes	Yes	Yes	Yes
7. Were the outcome measures prespecified, clearly defined, valid, reliable, and assessed consistently across all study participants?	Yes	Yes	Yes	Yes	Yes	Yes	Yes	Yes	Yes	Yes	Yes	Yes	Yes
8. Were the people assessing the outcomes blinded to the participants' exposures/interventions?	CD	CD	CD	P	CD	CD	CD	CD	CD	CD	CD	CD	CD

9. Was the loss to follow-up after baseline 20% or less? Were those lost to follow-up accounted for in the analysis?	NA	CD	CD	CD	Yes	CD	CD	CD	CD	CD	CD	CD	CD
10. Did the statistical methods examine changes in outcome measures from before to after the intervention? Were statistical tests done that provided p values for the pre-to-post changes?	Yes	Yes	Yes	Yes	Yes	Yes	Yes	Yes	Yes	Yes	Yes	Yes	Yes
11. Were outcome measures of interest taken multiple times before the intervention and multiple times after the intervention (i.e., did they use an interrupted time-series design)?	No	No	No	No	Yes	No	No	No	No	No	No	No	No
12. If the intervention was conducted at a group level (e.g., a whole hospital, a community, etc.) did the statistical analysis take into account the use of individual-level data to determine effects at the group level?	NA	NA	NA	NA	NA	NA	NA	NA	NA	NA	NA	NA	NA

Possible answers include: Yes, No, Not Applicable (NA), Not Reported (NR), Cannot Determine (CD) or Partial (P). For instructions and details of quality assessment tool see: https://www.nhlbi.nih.gov/health-topics/study-quality-assessment-tools

Table S9. Quality Assessment of Pre-Post Studies using NHLBI Quality Assessment Tool.

	Liu 2021[21]	Parksepp 2020[29]	Parksepp 2022[30]	Qiao 2016[31]	Suvitaival 2016[33]	Tkachev 2021[35]	Wang 2018[36]	Wang 2022[37]	Xuan 2011[39]	Yan 2018[40]	Yao 2010a[41]	Yao 2010b[42]
**1. Was the study question or objective clearly stated?**	Yes	Yes	Yes	Yes	Yes	Yes	Yes	Yes	Yes	Yes	Yes	Yes
**2. Were eligibility/selection criteria for the study population prespecified and clearly described?**	Yes	Yes	Yes	Yes	Yes	Yes	Yes	Yes	Yes	Yes	Yes	Yes
**3. Were the participants in the study representative of those who would be eligible for the test/service/intervention in the general or clinical population of interest?**	Yes	Yes	Yes	Yes	Yes	Yes	Yes	Yes	Yes	Yes	Yes	Yes
**4. Were all eligible participants that met the prespecified entry criteria enrolled?**	Yes	No	Yes	Yes	Yes	Yes	Yes	Yes	Yes	Yes	Yes	Yes
**5. Was the sample size sufficiently large to provide confidence in the findings?**	No	No	Yes	No	No	No	No	No	No	No	No	No
**6. Was the test/service/intervention clearly described and delivered consistently across the study population?**	Yes	Yes	Yes	Yes	Yes	Yes	Yes	Yes	Yes	Yes	Yes	Yes
**7. Were the outcome measures prespecified, clearly defined, valid, reliable, and assessed consistently across all study participants?**	Yes	Yes	Yes	Yes	Yes	Yes	Yes	Yes	Yes	Yes	Yes	Yes
**8. Were the people assessing the outcomes blinded to the participants' exposures/interventions?**	CD	CD	CD	Yes	CD	CD	CD	CD	CD	CD	CD	CD
**9. Was the loss to follow-up after baseline 20% or less? Were those lost to follow-up accounted for in the analysis?**	CD	CD	Yes	CD	CD	CD	CD	CD	CD	CD	CD	CD
**10. Did the statistical methods examine changes in outcome measures from before to after the intervention? Were statistical tests done that provided p values for the pre-to-post changes?**	Yes	Yes	Yes	Yes	Yes	Yes	Yes	Yes	Yes	Yes	Yes	Yes
**11. Were outcome measures of interest taken multiple times before the intervention and multiple times after the intervention (i.e., did they use an interrupted time-series design)?**	No	No	No	No	No	No	No	No	No	No	No	No

**12. If the intervention was conducted at a group level (e.g., a whole hospital, a community, etc.) did the statistical analysis take into account the use of individual-level data to determine effects at the group level?**	NA	NA	NA	NA	NA	NA	NA	NA	NA	NA	NA	NA

Possible answers include: Yes, No, Not Applicable (NA), Not Reported (NR), Cannot Determine (CD) or Partial (P). For instructions and details of quality assessment tool see: https://www.nhlbi.nih.gov/health-topics/study-quality-assessment-tools

Table S10. Quality Assessment of Controlled Intervention Studies using NHLBI Quality Assessment Tool.

	McEvoy 2013[23]
1. Was the study described as randomized, a randomized trial, a randomized clinical trial, or an RCT?	Yes
2. Was the method of randomization adequate (i.e., use of randomly generated assignment)?	Yes
3. Was the treatment allocation concealed (so that assignments could not be predicted)?	Yes
4. Were study participants and providers blinded to treatment group assignment?	Yes
5. Were the people assessing the outcomes blinded to the participants' group assignments?	Yes
6. Were the groups similar at baseline on important characteristics that could affect outcomes (e.g., demographics, risk factors, co-morbid conditions)?	Yes
7. Was the overall drop-out rate from the study at endpoint 20% or lower of the number allocated to treatment?	Yes
8. Was the differential drop-out rate (between treatment groups) at endpoint 15 percentage points or lower?	Yes
9. Was there high adherence to the intervention protocols for each treatment group?	NR
10. Were other interventions avoided or similar in the groups (e.g., similar background treatments)?	Yes
11. Were outcomes assessed using valid and reliable measures, implemented consistently across all study participants?	Yes
12. Did the authors report that the sample size was sufficiently large to be able to detect a difference in the main outcome between groups with at least 80% power?	NR
13. Were outcomes reported or subgroups analyzed prespecified (i.e., identified before analyses were conducted)?	Yes
14. Were all randomized participants analyzed in the group to which they were originally assigned, i.e., did they use an intention-to-treat analysis?	Yes

Possible answers include: Yes, No, Not Applicable (NA), Not Reported (NR), Cannot Determine (CD) or Partial (P). For instructions and details of quality assessment tool see: https://www.nhlbi.nih.gov/health-topics/study-quality-assessment-tools

REFERENCES

1. Al Awam, K.; Haußleiter, I.S.; Dudley, E.; Donev, R.; Brüne, M.; Juckel, G.; Thome, J. Multiplatform metabolome and proteome profiling identifies serum metabolite and protein signatures as prospective biomarkers for schizophrenia. *J Neural Transm (Vienna)* **2015**, *122 Suppl 1*, S111-122, doi:10.1007/s00702-014-1224-0.
2. Aquino, A.; Alexandrino, G.L.; Guest, P.C.; Augusto, F.; Gomes, A.F.; Murgu, M.; Steiner, J.; Martins-de-Souza, D. Blood-Based Lipidomics Approach to Evaluate Biomarkers Associated With Response to Olanzapine, Risperidone, and Quetiapine Treatment in Schizophrenia Patients. *Frontiers in psychiatry* **2018**, *9*, 209-209, doi:10.3389/fpsyt.2018.00209.
3. Atagün, M.İ.; Şıkoğlu, E.M.; Can, S.S.; Uğurlu, G.K.; Kaymak, S.U.; Çayköylü, A.; Algın, O.; Phillips, M.L.; Moore, C.M.; Öngür, D. Neurochemical differences between bipolar disorder type I and II in superior temporal cortices: A proton magnetic resonance spectroscopy study. *Journal of affective disorders* **2018**, *235*, 15-19, doi:10.1016/j.jad.2018.04.010.
4. Bicikova, M.; Hill, M.; Ripova, D.; Mohr, P.; Hampl, R. Determination of steroid metabolome as a possible tool for laboratory diagnosis of schizophrenia. *J Steroid Biochem Mol Biol* **2013**, *133*, 77-83, doi:10.1016/j.jsbmb.2012.08.009.
5. Brunkhorst-Kanaan, N.; Klatt-Schreiner, K.; Hackel, J.; Schröter, K.; Trautmann, S.; Hahnefeld, L.; Wicker, S.; Reif, A.; Thomas, D.; Geisslinger, G.; et al. Targeted lipidomics reveal derangement of ceramides in major depression and bipolar disorder. *Metabolism: clinical and experimental* **2019**, *95*, 65-76, doi:10.1016/j.metabol.2019.04.002.
6. Buretić-Tomljanović, A.; Giacometti, J.; Nadalin, S.; Rubesa, G.; Vulin, M.; Tomljanović, D. Phospholipid membrane abnormalities and reduced niacin skin flush response in schizophrenia. *Psychiatria Danubina* **2008**, *20*, 372-383.
7. Cai, H.L.; Li, H.D.; Yan, X.Z.; Sun, B.; Zhang, Q.; Yan, M.; Zhang, W.Y.; Jiang, P.; Zhu, R.H.; Liu, Y.P.; et al. Metabolomic analysis of biochemical changes in the plasma and urine of first-episode neuroleptic-naïve schizophrenia patients after treatment with risperidone. *Journal of proteome research* **2012**, *11*, 4338-4350, doi:10.1021/pr300459d.
8. Cao, B.; Jin, M.; Brietzke, E.; McIntyre, R.S.; Wang, D.; Rosenblat, J.D.; Ragguett, R.-M.; Zhang, C.; Sun, X.; Rong, C.; et al. Serum metabolic profiling using small molecular water-soluble metabolites in individuals with schizophrenia: A longitudinal study using a pre–post-treatment design. *Psychiatry and clinical neurosciences* **2019**, *73*, 100-108, doi:10.1111/pcn.12779.
9. Condray, R.; Dougherty, G.G.; Keshavan, M.S.; Reddy, R.D.; Haas, G.L.; Montrose, D.M.; Matson, W.R.; McEvoy, J.; Kaddurah-Daouk, R.; Yao, J.K. 3-Hydroxykynurenine and clinical symptoms in first-episode neuroleptic-naive patients with schizophrenia. *The international journal of neuropsychopharmacology / official scientific journal of the Collegium Internationale Neuropsychopharmacologicum (CINP)* **2011**, *14*, 756-767, doi:10.1017/S1461145710001689.
10. de Almeida, V.; Alexandrino, G.L.; Aquino, A.; Gomes, A.F.; Murgu, M.; Dobrowolny, H.; Guest, P.C.; Steiner, J.; Martins-de-Souza, D. Changes in the blood plasma lipidome associated with effective or poor response to atypical antipsychotic treatments in schizophrenia patients. *Progress in Neuro-Psychopharmacology and Biological Psychiatry* **2020**, *101*, 109945, doi:https://doi.org/10.1016/j.pnpbp.2020.109945.
11. Evans, S.J.; Ringrose, R.N.; Harrington, G.J.; Mancuso, P.; Burant, C.F.; McInnis, M.G. Dietary intake and plasma metabolomic analysis of polyunsaturated fatty acids in bipolar subjects reveal dysregulation of linoleic acid metabolism. *Journal of psychiatric research* **2014**, *57*, 58-64, doi:10.1016/j.jpsychires.2014.06.001.

12. Fujii, T.; Hattori, K.; Miyakawa, T.; Ohashi, Y.; Sato, H.; Kunugi, H. Metabolic profile alterations in the postmortem brains of patients with schizophrenia using capillary electrophoresis-mass spectrometry. *Schizophrenia research* **2017**, *183*, 70-74, doi:10.1016/j.schres.2016.11.011.
13. He, Y.; Yu, Z.; Giegling, I.; Xie, L.; Hartmann, A.M.; Prehn, C.; Adamski, J.; Kahn, R.; Li, Y.; Illig, T.; et al. Schizophrenia shows a unique metabolomics signature in plasma. *Translational psychiatry* **2012**, *2*, e149, doi:10.1038/tp.2012.76.
14. Kaddurah-Daouk, R.; McEvoy, J.; Baillie, R.A.; Lee, D.; Yao, J.K.; Doraiswamy, P.M.; Krishnan, K.R. Metabolomic mapping of atypical antipsychotic effects in schizophrenia. *Molecular psychiatry* **2007**, *12*, 934-945, doi:10.1038/sj.mp.4002000.
15. Kim, S.; Okazaki, S.; Otsuka, I.; Shinko, Y.; Horai, T.; Shimmyo, N.; Hirata, T.; Yamaki, N.; Tanifuji, T.; Boku, S.; et al. Searching for biomarkers in schizophrenia and psychosis: Case-control study using capillary electrophoresis and liquid chromatography time-of-flight mass spectrometry and systematic review for biofluid metabolites. *Neuropsychopharmacology Reports* **2022**, *42*, 42-51, doi:10.1002/npr2.12223.
16. Kriisa, K.; Leppik, L.; Balõtšev, R.; Ottas, A.; Soomets, U.; Koido, K.; Volke, V.; Innos, J.; Haring, L.; Vasar, E.; et al. Profiling of Acylcarnitines in First Episode Psychosis before and after Antipsychotic Treatment. *Journal of proteome research* **2017**, *16*, 3558-3566, doi:10.1021/acs.jproteome.7b00279.
17. Lenski, M.; Sidibé, J.; Gholam, M.; Hennart, B.; Dubath, C.; Augsburger, M.; von Gunten, A.; Conus, P.; Allorge, D.; Thomas, A.; et al. Metabolomic alteration induced by psychotropic drugs: Short-term metabolite profile as a predictor of weight gain evolution. *Clinical and translational science* **2021**, *14*, 2544-2555, doi:10.1111/cts.13122.
18. Li, N.; Yang, P.; Tang, M.; Liu, Y.; Guo, W.; Lang, B.; Wang, J.; Wu, H.; Tang, H.; Yu, Y.; et al. Reduced erythrocyte membrane polyunsaturated fatty acid levels indicate diminished treatment response in patients with multi- versus first-episode schizophrenia. *npj Schizophrenia* **2022**, *8*, doi:10.1038/s41537-022-00214-2.
19. Liu, M.L.; Zhang, X.T.; Du, X.Y.; Fang, Z.; Liu, Z.; Xu, Y.; Zheng, P.; Xu, X.J.; Cheng, P.F.; Huang, T.; et al. Severe disturbance of glucose metabolism in peripheral blood mononuclear cells of schizophrenia patients: a targeted metabolomic study. *J Transl Med* **2015**, *13*, 226, doi:10.1186/s12967-015-0540-y.
20. Liu, D.; An, Z.L.; Li, P.F.; Chen, Y.H.; Zhang, R.P.; Liu, L.H.; He, J.M.; Abliz, Z. A targeted neurotransmitter quantification and nontargeted metabolic profiling method for pharmacometabolomics analysis of olanzapine by using UPLC-HRMS. *RSC ADVANCES* **2020**, *10*, 18305-18314, doi:10.1039/d0ra02406f.
21. Liu, J.H.; Chen, N.; Guo, Y.H.; Guan, X.N.; Wang, J.; Wang, D.; Xiu, M.H. Metabolomics-based understanding of the olanzapine-induced weight gain in female first-episode drug-naïve patients with schizophrenia. *Journal of psychiatric research* **2021**, *140*, 409-415, doi:10.1016/j.jpsychires.2021.06.001.
22. Maes, M.; Landucci Bonifacio, K.; Morelli, N.R.; Vargas, H.O.; Barbosa, D.S.; Carvalho, A.F.; Nunes, S.O.V. Major Differences in Neurooxidative and Neuronitrosative Stress Pathways Between Major Depressive Disorder and Types I and II Bipolar Disorder. *Molecular neurobiology* **2019**, *56*, 141-156, doi:10.1007/s12035-018-1051-7.
23. McEvoy, J.; Baillie, R.A.; Zhu, H.; Buckley, P.; Keshavan, M.S.; Nasrallah, H.A.; Dougherty, G.G.; Yao, J.K.; Kaddurah-Daouk, R. Lipidomics reveals early metabolic changes in subjects with schizophrenia: effects of atypical antipsychotics. *PloS one* **2013**, *8*, e68717-e68717, doi:10.1371/journal.pone.0068717.

24. Mednova, I.A.; Chernonosov, A.A.; Kasakin, M.F.; Kornetova, E.G.; Semke, A.V.; Bokhan, N.A.; Koval, V.V.; Ivanova, S.A. Amino Acid and Acylcarnitine Levels in Chronic Patients with Schizophrenia: A Preliminary Study. *Metabolites* **2021**, *11*, doi:10.3390/metabo11010034.
25. Mednova, I.A.; Chernonosov, A.A.; Kornetova, E.G.; Semke, A.V.; Bokhan, N.A.; Koval, V.V.; Ivanova, S.A. Levels of Acylcarnitines and Branched-Chain Amino Acids in Antipsychotic-Treated Patients with Paranoid Schizophrenia with Metabolic Syndrome. *Metabolites* **2022**, *12*, doi:10.3390/metabo12090850.
26. Okamoto, N.; Ikenouchi, A.; Watanabe, K.; Igata, R.; Fujii, R.; Yoshimura, R. A Metabolomics Study of Serum in Hospitalized Patients With Chronic Schizophrenia. *Frontiers in psychiatry* **2021**, *12*, 763547, doi:10.3389/fpsyt.2021.763547.
27. Orešič, M.; Tang, J.; Seppänen-Laakso, T.; Mattila, I.; Saarni, S.E.; Saarni, S.I.; Lönnqvist, J.; Sysi-Aho, M.; Hyötyläinen, T.; Perälä, J.; et al. Metabolome in schizophrenia and other psychotic disorders: A general population-based study. *Genome medicine* **2011**, *3*, doi:10.1186/gm233.
28. Paredes, R.M.; Quinones, M.; Marballi, K.; Gao, X.; Valdez, C.; Ahuja, S.S.; Velligan, D.; Walss-Bass, C. Metabolomic profiling of schizophrenia patients at risk for metabolic syndrome. *The international journal of neuropsychopharmacology / official scientific journal of the Collegium Internationale Neuropsychopharmacologicum (CINP)* **2014**, *17*, 1139-1148, doi:10.1017/S1461145714000157.
29. Parksepp, M.; Leppik, L.; Koch, K.; Uppin, K.; Kangro, R.; Haring, L.; Vasar, E.; Zilmer, M. Metabolomics approach revealed robust changes in amino acid and biogenic amine signatures in patients with schizophrenia in the early course of the disease. *Scientific reports* **2020**, *10*, 13983, doi:10.1038/s41598-020-71014-w.
30. Parksepp, M.; Haring, L.; Kilk, K.; Koch, K.; Uppin, K.; Kangro, R.; Zilmer, M.; Vasar, E. The Expanded Endocannabinoid System Contributes to Metabolic and Body Mass Shifts in First-Episode Schizophrenia: A 5-Year Follow-Up Study. *Biomedicines* **2022**, *10*, doi:10.3390/biomedicines10020243.
31. Qiao, Y.; Zhang, L.; He, S.; Wen, H.; Yu, Y.M.; Cao, C.H.; Li, H.F. Plasma metabonomics study of first-Episode schizophrenia treated with olanzapine in female patients. *Neuroscience letters* **2016**, *617*, 270-276, doi:10.1016/j.neulet.2016.02.031.
32. Schwarz, E.; Prabakaran, S.; Whitfield, P.; Major, H.; Leweke, F.M.; Koethe, D.; McKenna, P.; Bahn, S. High Throughput Lipidomic Profiling of Schizophrenia and Bipolar Disorder Brain Tissue Reveals Alterations of Free Fatty Acids, Phosphatidylcholines, and Ceramides. *Journal of proteome research* **2008**, *7*, 4266-4277, doi:10.1021/pr800188y.
33. Suvitaival, T.; Mantere, O.; Kieseppä, T.; Mattila, I.; Pöhö, P.; Hyötyläinen, T.; Suvisaari, J.; Orešič, M. Serum metabolite profile associates with the development of metabolic co-morbidities in first-episode psychosis. *Translational psychiatry* **2016**, *6*, e951-e951, doi:10.1038/tp.2016.222.
34. Tessier, C.; Sweers, K.; Frajerman, A.; Bergaoui, H.; Ferreri, F.; Delva, C.; Lapidus, N.; Lamaziere, A.; Roiser, J.P.; De Hert, M.; et al. Membrane lipidomics in schizophrenia patients: a correlational study with clinical and cognitive manifestations. *Translational psychiatry* **2016**, *6*, e906-e906, doi:10.1038/tp.2016.142.
35. Tkachev, A.; Stekolshchikova, E.; Anikanov, N.; Zozulya, S.; Barkhatova, A.; Klyushnik, T.; Petrova, D. Shorter chain triglycerides are negatively associated with symptom improvement in schizophrenia. *Biomolecules* **2021**, *11*, doi:10.3390/biom11050720.
36. Wang, D.; Sun, X.; Yan, J.; Ren, B.; Cao, B.; Lu, Q.; Liu, Y.; Zeng, J.; Huang, N.; Xie, Q.; et al. Alterations of eicosanoids and related mediators in patients with schizophrenia. *Journal of psychiatric research* **2018**, *102*, 168-178, doi:10.1016/j.jpsychires.2018.04.002.
37. Wang, X.; Xiu, M.; Wang, K.; Su, X.; Li, X.; Wu, F. Plasma linoelaidyl carnitine levels positively correlated with symptom improvement in olanzapine-treated first-episode drug-naïve

schizophrenia. *Metabolomics : Official journal of the Metabolomic Society* **2022**, *18*, 50, doi:10.1007/s11306-022-01909-4.
38. Wood, P.L.; Unfried, G.; Whitehead, W.; Phillipps, A.; Wood, J.A. Dysfunctional plasmalogen dynamics in the plasma and platelets of patients with schizophrenia. *Schizophrenia research* **2015**, *161*, 506-510, doi:10.1016/j.schres.2014.11.032.
39. Xuan, J.; Pan, G.; Qiu, Y.; Yang, L.; Su, M.; Liu, Y.; Chen, J.; Feng, G.; Fang, Y.; Jia, W.; et al. Metabolomic Profiling to Identify Potential Serum Biomarkers for Schizophrenia and Risperidone Action. *Journal of proteome research* **2011**, *10*, 5433-5443, doi:10.1021/pr2006796.
40. Yan, L.; Zhou, J.; Wang, D.; Si, D.; Liu, Y.; Zhong, L.; Yin, Y. Unbiased lipidomic profiling reveals metabolomic changes during the onset and antipsychotics treatment of schizophrenia disease. *Metabolomics : Official journal of the Metabolomic Society* **2018**, *14*, 80, doi:10.1007/s11306-018-1375-3.
41. Yao, J.K.; Dougherty, G.G.; Reddy, R.D.; Keshavan, M.S.; Montrose, D.M.; Matson, W.R.; McEvoy, J.; Kaddurah-Daouk, R. Homeostatic Imbalance of Purine Catabolism in First-Episode Neuroleptic-Naive Patients with Schizophrenia. *PloS one* **2010**, *5*, doi:10.1371/journal.pone.0009508.
42. Yao, J.K.; Dougherty, G.G., Jr.; Reddy, R.D.; Keshavan, M.S.; Montrose, D.M.; Matson, W.R.; Rozen, S.; Krishnan, R.R.; McEvoy, J.; Kaddurah-Daouk, R. Altered interactions of tryptophan metabolites in first-episode neuroleptic-naive patients with schizophrenia. *Molecular psychiatry* **2010**, *15*, 938-953, doi:10.1038/mp.2009.33.
